# Alignment behaviors of short peptides provide a roadmap for functional profiling of metagenomic data

**DOI:** 10.1186/s12864-015-2272-z

**Published:** 2015-12-21

**Authors:** Rohita Sinha, Jennifer Clarke, Andrew K. Benson

**Affiliations:** Department of Food Science and Technology, University of Nebraska, 256 Food Innovation Complex, Lincoln, NE 68588-6205 USA; Department of Statistics, University of Nebraska, Lincoln, NE 68583 USA; Quantitative Life Sciences Initiative, University of Nebraska, Lincoln, NE 68583 USA

## Abstract

**Background:**

Functional assignments for short-read metagenomic data pose a significant computational challenge due to perceived unpredictability of alignment behavior and the inability to infer useful functional information from translated protein-fragments/peptides. To address this problem, we have examined the predictability of short peptide alignments by systematically studying alignment behavior of large sets of short peptides generated from well-characterized proteins as well as hypothetical proteins in the KEGG database.

**Results:**

Using test sets of peptides modeling the length and phylogenetic distributions of short-read metagenomic data, we observed that peptides from well-characterized proteins had indistinguishable alignments to proteins from the same orthologous family and proteins from different families. Nonetheless, the patterns contained remarkable phylogenetic and structural signals, with alignments of even very short peptides naturally restricted to their orthologous family and/or proteins having similar structural folds. In stark contrast, peptides from “hypothetical proteins” had only sparse hit patterns with low frequencies and much lower identities. By weighting the structure-driven alignments and filtering peptides with behaviors similar to those derived from “hypothetical proteins”, we demonstrate that the accuracy of abundance predictions of protein families is dramatically improved.

**Conclusions:**

Evolutionary processes have dispersed protein folds across multiple protein families, precluding accurate functional assignment to short peptides, whose alignment behavior is non-random and driven by structure. Algorithms that filter sparse peptides and weight hit patterns of peptides from “known space” dramatically improve quantification of functions from diverse mixtures of peptides and should substantially improve applications of metagenomic analyses requiring accurate quantitative measures of functional families.

**Electronic supplementary material:**

The online version of this article (doi:10.1186/s12864-015-2272-z) contains supplementary material, which is available to authorized users.

## Background

Faster and economical next-generation DNA sequencing (NGS) technologies have enabled studies of complex microbial communities which were experimentally intractable in terms of their true microbial diversities only a decade ago [1–6]. Economy of scale and the availability of streamlined data processing pipelines have driven the majority of studies’ estimates of taxonomic and phylogenetic content from 16S ribosomal RNA sequencing and inferences of functional content from reference genomes of corresponding or related taxa. On the other hand, whole shotgun sequencing of metagenomic DNA arguably provides a more robust and unbiased measurement of the taxonomic and functional content of a microbiome [7, 8], but its use has been limited due to the necessity of greater sequencing depth (higher cost) and significant computational challenges. The latter is particularly acute, especially in non-human systems where genomic catalogues and reference genomes of representative species are not readily available. As sequencing costs continue to decline, the primary barrier for broad application of whole shotgun metagenome sequencing is largely computational.

In silico functional annotation of proteins exploits their evolutionary relationships with experimentally characterized proteins and uses empirically-defined thresholds of global sequence identity (e.g. > 40 %) to assign proteins to the same Enzyme Commission number (function) [9]. In the absence of such relationships, methods like I-TASSER [10] and COFACTOR [11] collectively annotate some protein sequences by predicting and comparing their structures with global and local structural features of well-characterized reference proteins. These powerful techniques, however, have been developed exclusively for full-length molecules, and use of similar approaches for peptides predicted from short-read metagenomic data has generally been avoided due to the belief that such peptides lack enough evolutionary or structural information to accurately identify the orthologous genes from which they originate. These concerns are underscored by the fact that protein domains are redundantly used to perform diverse biochemical activities [12, 13], leading to the expectation that short peptides will simply align to all the proteins carrying their “domains of origin”, resulting in a confounded pattern of functional predictions based on a variety of reference proteins carrying that domain [14, 15].

The three prominent resources for metagenomic data processing (MEGAN [16], MG-RAST [17] and HUMAnN [18]) all work similarly, aligning translated peptides from the short reads of NGS platforms to databases of well-annotated reference proteins and using single sets of sequence similarity measures (SSMs) for functional prediction. The effectiveness of individual sets of SSMs used by these protocols was recently questioned by the finding of the PAUDA study [19], where high variances in the identity profiles of alignment hits were observed even within the same KEGG-orthology group (KO) [20]. These observations resulted in concerns of significant sensitivity losses in assigning KO-families to short NGS reads on the basis of individual sets of SSMs. Moreover, recent publications using these metagenomic data processing methods also demonstrate absence of any consensus among the community of users regarding individual significance thresholds or sets of SSMs elements that can accurately discriminate between true and false-positive function assignments [21–26].

Given the dearth of empirically-derived data on the alignment behavior of peptides that could even be used to model thresholds for SSMs, we were motivated to systematically study the actual alignment behavior of short protein fragments. Using random peptides extracted from KO-family members (the “known” protein universe) and hypothetical uncharacterized proteins (the “unknown” protein universe) extracted from the M5nr database [27], we studied their alignment behavior in bulk using bacterial proteins from the KO-families as a reference database. We observed remarkable behaviors that show clear evidence of structural features of local segments of proteins being evolutionarily constrained. These structural constraints act as natural barriers to random alignment of small peptides, restricting peptide alignments to homologous domains from the domain of origin and evolutionarily related families in which the domain has become associated with a new function. Peptides originating from uncharacterized/hypothetical proteins (“unknown” protein universe), which typically represent a significant part of the reads in metagenomic NGS data, do not display this characteristic alignment behavior and their parameters can be used as a filter to eliminate their confounding effects on abundance estimates of known protein families in metagenomic data.

## Results

To systematize measurements of alignment behavior, we developed sets of peptides from two major cross-sections of proteins that are observed in metagenomic data, namely, peptides from proteins that can be annotated accurately on the basis of experimentally-characterized protein families (Type 1 peptides/known protein universe) and peptides that originate from proteins that share no detectable evolutionary relationship with known protein families (Type 2 peptides/unknown protein universe). For Type 1 peptides, eight different sets of peptides were generated from the KO-families with each set having a different peptide length (range 11aa to 81aa and termed Type1_11aa-Type1_81aa, respectively), see Methods and Table 2 for the test case types description. Each set contained 3 randomly-derived peptides from each of the 6327 KO-families comprising our reference database. These sets were then aligned to the entire set of bacterial members of the 6327 KOs. Viewing the alignments as a whole (Additional file 1: Figure S1–8), the longer query peptides generally yielded a higher frequency of significant hits. With the smallest query peptide length tested (11-mers), only 22.1 % found a significant hit when the e-value cutoff was 10 (default BLASTP cutoff). Increasing the peptide length dramatically increases the frequency of significant hits, with the highest frequency of significant hits equal to the length of the query peptide (Additional file 1: Figure S1–8). Very similar behavior was observed when e-values were plotted for the different query peptide lengths (Additional file 1: Figure S9–16).

**Fig. 1:**
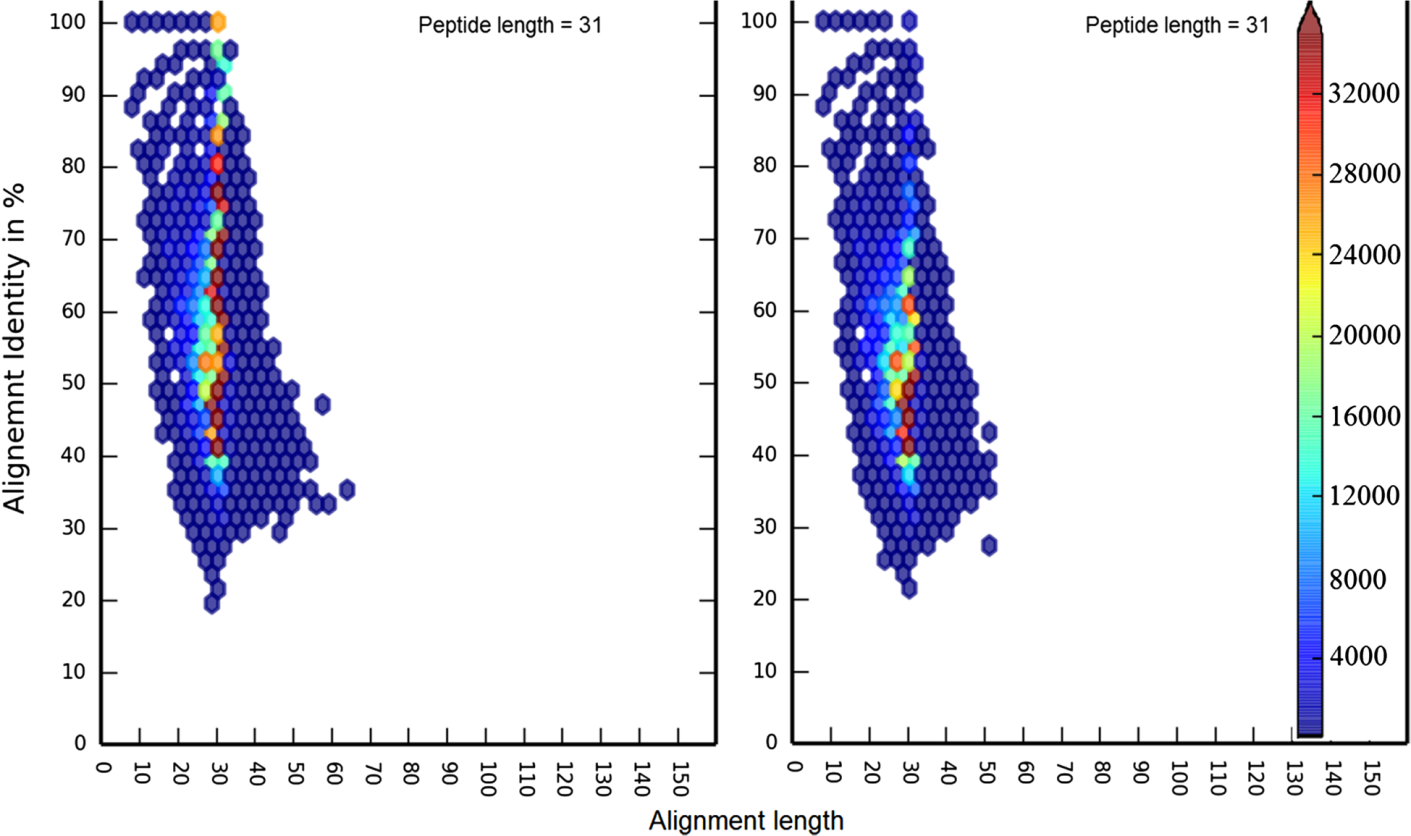
Alignment profiles of short peptides to parental vs non-parental KO-families. Comparison of alignment behavior (*Left panel*) when the short peptides align to members of their parent KO-families (*Right panel*) when short peptides align to members of their non-parent KO-families. While hits to same KO-family members have high proportion of alignment-identity above 80 %, but a major fraction of hits still remain in the range of 40–80 % (for both left & right panel) and makes it difficult to discriminate between true and false positive hits. Hexbin colors within the graph are proportional to their frequency or members within the bin. Member frequency and color relationship is depicted in the arrow-headed color bar

**Fig. 2:**
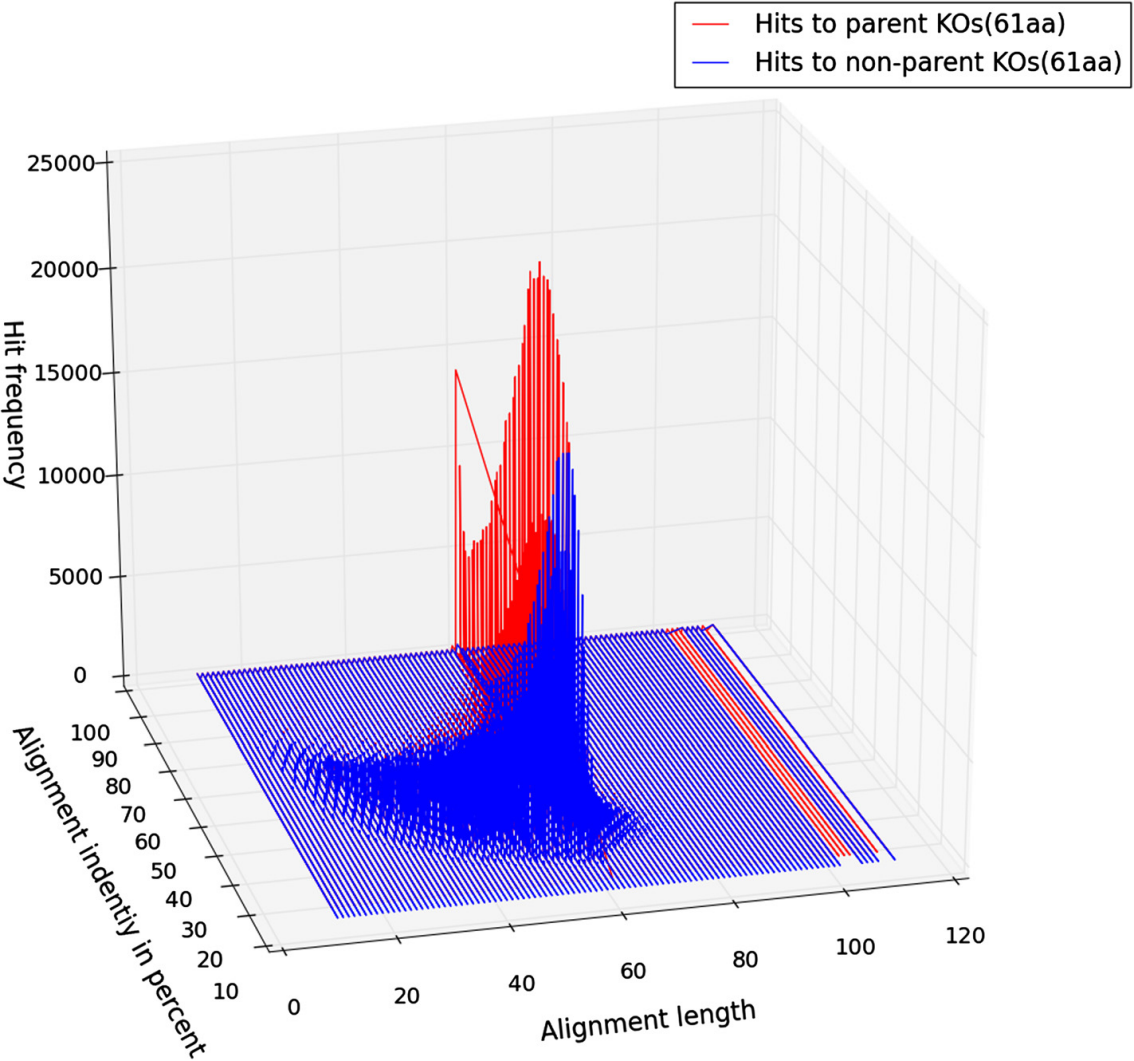
Three dimensional plot of alignment length, percent identity and hit frequency of type 1 peptides. The plot is colored to differentiate values for hits to parental KO (*red*) and non-parental KO-family (*blue*). Data for the 61-mer peptides is shown

Because the origin of the query peptides was known, their alignment behaviors to proteins of the parental KO-family (the same KO-family from which the peptide originated) and of the non-parental KO-families (all other KO-families excluding the parental KO) could be quantified independently, as depicted in (Fig. 1) for query peptides of length 31 amino acids. When comparing the alignment patterns of the queries to parental and non-parental KO-families, the behaviors were very consistent irrespective of the length of the query peptides (Type1_11-81aa). To statistically confirm this phenomenon we performed one way ANOVA over differences of alignment-identity values, between parental and non-parental KOs, of 500 randomly-selected hits from each peptide length (21–81, length 11 is not considered since >90 % of the hits were within ~100 % identity range). The mean p-value (0.1127) over 100 iterations of this procedure clearly shows that the alignment behavior patterns were independent of the length of the peptides. We noticed that alignments with higher percent identity (Additional file 1: Figure S1–8) or lower e-values (Additional file 1: Figure S9–16) were more frequently observed among alignments to parental KO-families than non-parental KO-families. Box and whisker plots of the distributions of percent identity of full-length alignments of each of the query peptide lengths (Additional file 1: Figure S17) shows that the majority of alignments to parental KOs consistently occur at a higher range of identities than alignments to non-parental KOs. However, a more accurate picture emerges when alignments of all lengths are considered. In three dimensions (alignment length, percent identity and frequency) there is substantial overlap of hits to parental and non-parental KOs (Fig. 2 and Additional file 1: Figure S18–23). The overlap of the three-dimensional contours suggests that no single threshold would easily discriminate between the parental and non-parental KO contours and hence allow consistent resolution of parental and non-parental KO-families. These observations were further strengthened by the analysis of multiple ROC curves (Additional file 1: Figure S24) generated for multiple peptide lengths (31, 51 and 71aa) with varying range of alignment parameters (alignment coverage and identity levels). These plots (Additional file 1: Figure S24) clearly indicate that none of the combinations of alignment-coverage and identity provided high enough sensitivity (true positive rate) and specificity (true negative rate) to accurately assign short peptides to KO-families.

Though the lengths of the most abundant alignments were equivalent to the length of the query peptide, we observed some alignments as long as twice the length of the query peptide and such behavior was equally prevalent among hits to parental as well as to non-parental KO-families (Additional file 1: Figure S25). Interestingly the hits to non-parental KO-families show similar alignment patterns, hinting that structural and functional similarity may be shared among alignments to non-parental KOs and parental KO-families (further explored below in the section “Why do hits to non-parental KO-families have competitive alignments”).

### Error rate using only the best alignment

Because the alignment behavior of type 1 peptides precludes a simple discrimination between parental and non-parental KOs, we estimated the error rate of classification for peptides when an orthologue from a very closely related species is *not* present in the database. This was estimated by the fraction of peptides (from Type1_11-81aa set) having their best hits with a protein from a different (non-parental) KO-family, even when the members of their own KO-families were present in the reference database (self-hit is not considered). Table 1 shows that ~91 % of the peptides of each query length found their best hit among parental KOs (last two columns), while the remaining 9 % of the peptides had their best-hits among non-parental KOs. This ratio of true-positive/false-positive was essentially independent of query peptide length and suggests that nearly 10 % of assignments based on best-hits may be incorrect.

**Table 1:** Alignment behavior of short-peptides (peptide length 11aa–81aa)

Peptide Length	Total peptides^a^	Fraction aligned^b^	Total number of blast-hits^c^	Total aligned to same KO (%)^d^	Total aligned to different KO (%)^e^	Total of best-hits aligned to same KO (%)^f^	Total of best-hits aligned to different KO (%)^g^
11	18,981	4196 (22.1)	57,705	48,677 (84.3)	9028 (15.6)	3964 (94.4)	232 (5.6)
21	18,978	16,482 (86.8)	1,200,295	815,915 (67.9)	384,380 (32.0)	15,366 (93.2)	1116 (6.8)
31	18,960	18,104 (95.4)	2,348,640	1,409,131 (59.9)	939,509 (40.0)	16,449 (90.8)	1655 (9.2)
41	18,900	18,626 (98.5)	3,181,987	1,784,051 (56.0)	1,397,936 (43.9)	16,855 (90.5)	1771 (9.5)
51	18,807	18,728 (99.5)	3,829,200	2,050,119 (53.5)	1,779,081 (46.4)	16,912 (90.3)	1816 (9.7)
61	18,723	18,701 (99.8)	4,266,683	2,199,736 (51.5)	2,066,947 (48.4)	16,986 (90.8)	1715 (9.2)
71	18,564	18,554 (99.9)	4,598,168	2,313,719 (50.0)	2,284,449 (49.6)	16,907 (91.1)	1647 (8.9)
81	18,396	18,391 (99.9)	4,839,697	2,387,627 (49.3)	2,452,070 (50.6)	16,765 (91.1)	1626 (8.9)

^a^Total number of short-peptides used in the study

^b^Total fraction of peptides having significant alignment with at least one other protein (self-hits are not considered)

^c^Total count of significant BLAST hits

^d^Total count of significant BLAST hits to the same KO group (percentage)

^e^Total count of significant BLAST hits to a different KO group (percentage)

^f^Total count of best BLAST hits aligning to the same KO group (percentage)

^g^Total count of best BLAST hits aligning to a different KO group (percentage)

### Total number of members within a KO-family influences the quality of true-positive alignments

Despite the ever-increasing number of diverse microbial taxa whose genomes have been sequenced and carefully annotated, even the most carefully curated databases such as KEGG have unequal representation across taxonomic and phylogenetic space and consequently have corresponding overrepresentation and voids in functional ontologies. Given the broad distributions of percent identities, alignment lengths and e-values for alignments of type 1 peptides, it seems reasonable to expect that biases in the databases affect these distributions, further confounding assignments based on alignment alone. To model the effects of database bias, the relationships between the number of KO-family members in the database and the median alignment identity of all true positive hits of peptides were plotted. The plots (Additional file 1: Figure S26–33) revealed that the range of alignment identities was quite large for KOs with fewer members, but got much smaller as the KO size increased, with median alignment identity decreasing as KO-family size increases. Thus, KO-families with higher representation may cover a larger evolutionary space of proteins but the effect is to lower the median score and tighten the distribution of the identities, while the identities at low KO representation are much more dispersed with an inflated median. This trend was independent of query peptide length, although the rate of decrease in the median identity score increased with longer peptide length. This is likely because longer peptides have a higher probability of having medium quality hits that are long enough to cross the BLAST threshold score.

### Why do hits to non-parental KO-families have competitive alignments?

Though database representation clearly affects alignment distributions and confounds the parental KO/non-parental KO boundary on the alignment behavior landscape, another factor that likely affects the landscape is structural divergence. Protein fold space is limited [28, 29] and the same folds are often found in proteins that perform quite different biochemical functions [12]. In contrast, the reverse (convergence of unique protein folds to execute the same function and sharing the same EC number) has occurred in only a small number of cases (7.5 % of all known EC nodes) [13]. Accordingly, proteins carrying similar EC-numbers (catalyzing similar reactions) have a high propensity of carrying similar domains/folds. We therefore hypothesized that the KO-families within the boundaries of the alignment landscapes of parental/non-parental KO-families share the same or highly related EC hierarchies.

This hypothesis was examined by querying the EC numbers of the peptides (from Type1_11-81aa) (Table 2) and the EC numbers of the ‘reference set’ (see Methods section) proteins to which they align and developing EC number similarity profiles at each level of the EC hierarchy. If our hypothesis is true, we would expect the EC numbers of the alignments to the non-parental KOs to increasingly match the EC numbers of the parental KOs at increasing levels of the EC taxonomy. As shown in Additional file 1: Figure S34, this is indeed the case, as query and aligning non-parental proteins generally shared EC hierarchies at frequency rates of ~80 % (1st level), 67 % (1st & 2nd level), 60 % (1st, 2nd and 3rd level) and finally 20 % across all four levels. Thus, even when peptide alignment identities are at the boundaries between parental and non-parental KOs, the functions performed by the peptide domains are similar and the the same was suggested by the alignment behavior of peptides to parental and non-parental KO-families. For example, aminoacyl-tRNA-synthetase proteins like aspartyl-tRNA-synthetase (K01876, EC:6.1.1.12) and lysyl-tRNA-synthetase (K04567, EC:6.1.1.6) have different KO-family assignments due to different ligand-specificity of their catalytic-domains but perform similar functions (similar EC-number profile up to the third level) by sharing the same anticodon binding domain (N-terminal *β*-barrel domain) to bind to tRNA [30].

**Table 2:** Description of test case notations used in the current study

Test case type	Description
Type1_11–81aa	Peptides were derived from well characterized proteins. In eight independent test cases lengths of peptides ranged between 11 to 81 amino-acids.
Type2.1–2.3	Peptides were derived from uncharacterized proteins and test cases were classified on the basis of the degree of sequence similarity of proteins with well-annotated proteins.
	2.1: Coverage <70 % & identity < 70 %
	2.2: Coverage <70 % & identity <50 %
	2.3: Coverage <70 % & identity <35 %
Type3	Simulated data to test the “Frequency weighted method”

Remarkably, our systematic characterization shows that despite being short in nature (11 to 81aa), the primary sequences of these short peptides carry information that reflects similarity in protein structure and function.

### Frequency weighted protein count method

The inherent structural constraints of proteins and the highly selective alignment of peptides to domains which are homologous to their parental KOs, even when present in non-parental KOs, means that artificial thresholding based on SSM constraints may actually limit the information that could be used for the function assignment. This is especially true given the effects of uneven KO representations in the databases. To correct for the uncertainty in the functional assignments of peptides having many significant alignments, rather than assigning a peptide to a single protein family we weight its contribution to total abundance values of all the protein families having significant alignment with that peptide. The alignment weights can be adjusted relative to the alignment weights of peptides which are highly specific to their parental KO-family (Frequency weight of peptide-X = 1/ Total number of significant alignments of peptide-X). This “frequency weighted read count” protocol provides higher weights to peptides unique to a protein family, and should improve the accuracy of protein abundance profiling by decreasing the noise created by reads with complex alignment patterns.

**Fig. 3:**
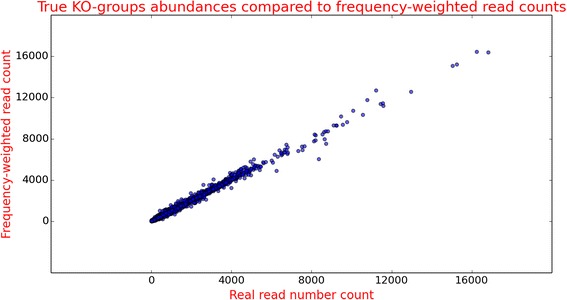
True KO-families abundances are compared with frequency-weighted read counts. Evaluation of performance of ‘Frequency weighted read count’ method when test case is comprised of peptides originating from those proteins, which have their family members in the ‘Reference set

To test our concept, 31-mer peptides were generated from all 6327 KO-families, randomly choosing about 10 % of the members from each KO-family, and these peptides were aligned against the complete ‘Reference set’ proteins (see Methods, “Test case Type 3” for details). In our protocol, the BLASTP outputs were first parsed to calculate and store the weight for each query peptide; once these weights were computed the abundance of each KO-family was calculated by adding the frequency-weight of all the peptides aligned to member proteins of the corresponding KO-family. The frequency-weight based read counts (abundance) were plotted against the true (unweighted) counts from the same KO-families from which the peptides originated (Fig. 3). Abundance values based on our “frequency-weighted read count” method achieve a very high correlation (Pearson correlation coefficient 0.99) with the true abundance values of all the KO-families present in our data set (details in Methods section).

### Alignment behavior of reads originating from experimentally uncharacterized proteins (Test case type2)

Even in some of the best studied bacterial species, significant proportions of the proteins fall into a category with no readily detectable evolutionary or structural relationship with experimentally characterized proteins. As an example of this category, 11 % of the proteins from E.coli do not show significant similarity with proteins of known structures, using even the most sophisticated threading algorithms to detect distant ancestry and predict functionality [31]. Such proteins have been designated as “hard” cases to assign structural folds or functions. Despite the predictable behavior of the full-length hard case proteins, it is quite possible that short fragments derived from hard case proteins can achieve significant alignment with well characterized proteins. Since a significant proportion of metagenomic data routinely falls into this hard case category, it is likely that alignment behavior of peptides from hard case proteins could confound quantification when using all alignment information of peptides in conjunction with frequency-based weighting.

**Fig. 4:**
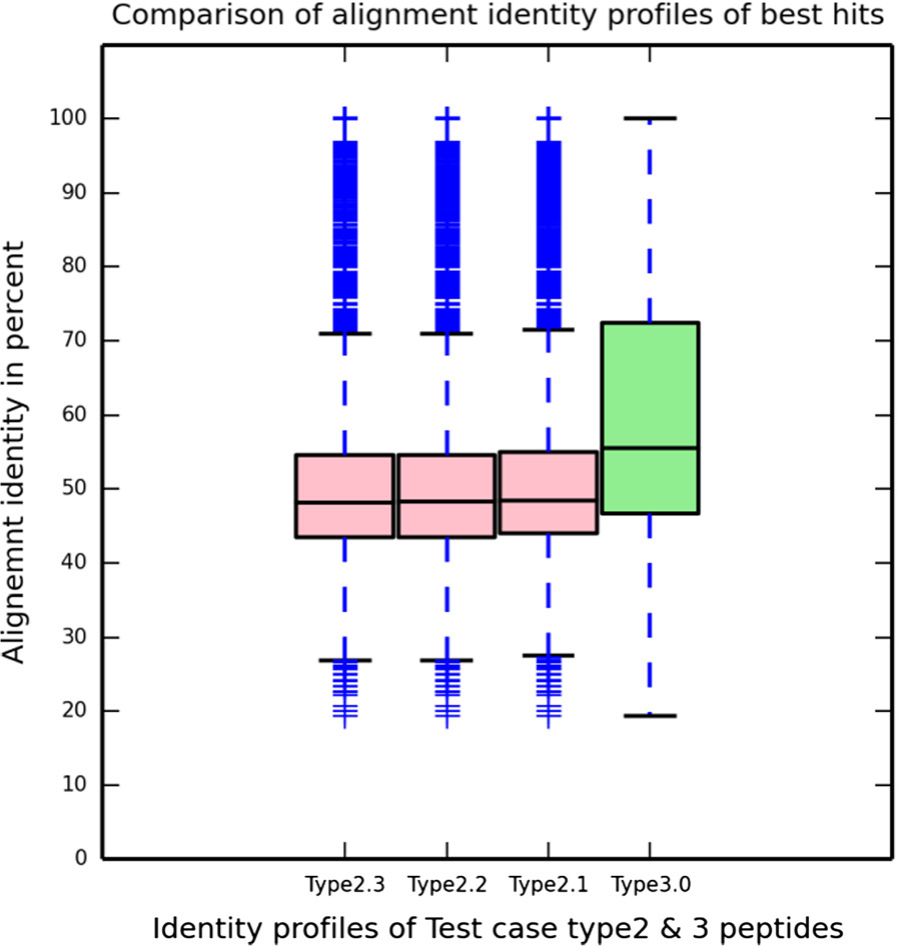
Comparison of alignment-identity profiles of the best hits of peptides from known and unknown protein-space. Identity profile of best hits of peptides from uncharacterized proteins (first three *pink* boxplots) is compared with the same of peptides from proteins having their family members in the reference protein set (*green*)

To study this potential confounder, we modeled the behavior of peptides derived hypothetical proteins using three different test sets of peptides (Type 2.1, 2.2 and 2.3) derived from hypothetical proteins of known genomes (see Methods section for details) that were aligned to the ‘Reference set proteins’. The alignment data (Additional file 1: Figure S27) showed several unique features that were not observed in the alignments to type 1 and type 3 peptides. First, only one-third of the type 2 peptides showed significant alignments (type 2.1 38 %, type 2.2 37 % and type 2.3 35 % compared to 99 % of type 1 and type 3 peptides). If the fraction of hypothetical proteins with at least one peptide having a significant hit with a reference protein is calculated, the numbers essentially doubled to 68, 68 and 67 % for Type 2.1, 2.2 and 2.3 test sets, respectively. Second, when compared to alignments of type 3 peptides studied above, type 2.1, 2.2 and 2.3 peptides had substantially fewer hits per peptide (3.74, 2.98 and 1.97, respectively) as compared to 200 hits/peptide for ‘Test set type 3’ peptides. Among the hits that were obtained, the type 2 peptides hit a very large proportion of the reference set (82.6 % for type 2.1, 79.5 % for type 2.2, and 72 % of type 2.3), showing the virtually random nature of these alignments. The randomness was also reflected in the percent identities of the best hits, which were far lower for the type 2 than for the type 3 peptides (Fig. 4).

Some ‘Test case type2’ peptides do achieve significant hits, even in the absence of homologous proteins in the reference set. It seems likely that that the sheer number of reads from genes of this category would affect quantification using our frequency-weighted method as these proteins are among the most commonly encountered in metagenomics data sets.

**Fig. 5:**
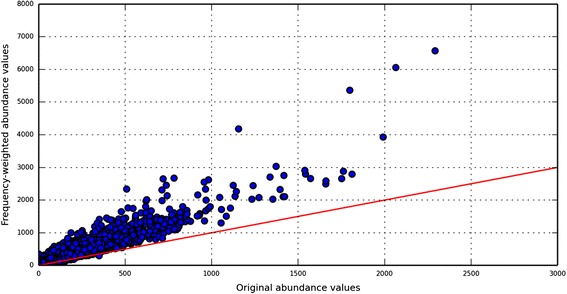
Effect of peptides from “unknown protein-space” on the “frequency-weighted” abundance profiles of proteins from “known space”. Artificial boost in the abundances of KO-families is elucidated using output of ‘Frequency weighted read count’ method when ‘Test case type2.3’ (peptides from unknown space) is added to ‘Selected_4K_KO_Hits’. Red line reflects the true correspondence values

**Fig. 6:**
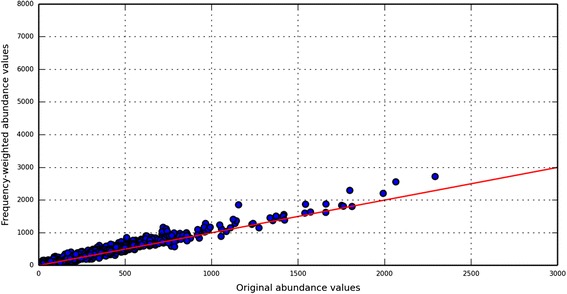
Corrected abundance profiles of KO-families using “Filter-enabled frequency weighted” method. Artificial boost in abundance values of KO-families due to peptides from “unknown protein space” is corrected by extending our frequency-weighted method and enable it to filter peptides with characteristics of those from hypothetical proteins. New abundance profile of the same test data used in Fig. 5 is plotted in this figure (the plot can be compared directly to the plot in Fig. 5). Red line reflects the true correspondence values

To measure their effect, the BLAST results of the ‘Test case type3’ dataset were pruned to hits of peptides from only 4000 randomly selected KO-families, referred to as the ‘Selected_4K_KO_Hits’. The frequency-weighted abundance profiles for all 6327 possible KO-families were then measured from only the ‘Selected_4K_KO_Hits’, or new sets in which the ‘Selected_4K_KO_Hits’ were composited with hits from peptides of the Test case type 2.1, 2.2 and 2.3. As expected, the ‘Selected_4K_KO_Hits’ alone showed a very high degree of correlation with their true abundance profiles (Additional file 1: Figure S36). In contrast, the massive numbers of hypothetical peptides in the composited ‘Selected_4K_KO_Hits’ plus type 2 peptides generated a large numbers of low per-peptide hits from the hypothetical proteins, inflating the abundances of many proteins substantially from their expected abundances (Fig. 5 and Additional file 1: Figure S37–39). To filter out the inflation from the random hits of hypothetical peptides we used two of their unique alignment behaviors, namely, their very low per-peptide hits (which ranges predominantly from 0 to 10 (Additional file 1: Figure S43–45)) and the low alignment-identity profiles from their best hits (median identity value of peptides from Test case 2 was ~55 % (Fig. 4)). Based on these patterns we revised our ‘Frequency weighted read count method’ to filter out or ignore the hits from those peptides which have (1) low per-peptide hit counts (<50 hits) and (2) a best hit with alignment identity below 55 %. Applying this new protocol to the sets of ‘Selected_4K_KO_Hits’ alone and the ‘Selected_4K_KO_Hits’ plus type 2 peptides (Fig. 6 and Additional file 1: Figure S40–42) shows that the effects of the type 2 peptides are mostly eliminated and the observed and expected abundances of the different KO-families show much greater correspondence. Collectively, incorporating systematic analyses of peptide alignment behavior into an approach to accurately assign functions results in more reliable quantification of gene abundances in metagenomic data sets.

## Discussion

Foundational studies of protein structure led to an early realization that structural information was influenced substantially by the sequence of the protein [32, 33]. The alignment behavior of short peptides was examined in detail by Sander and Schneider [34] and Rost [35] who showed that segments of proteins having alignment lengths between 10 and 80 aa are structural homologs provided the corresponding minimum alignment identities are 40–80 %. Our interest in further examining this behavior has been renewed by the capacity to explore taxonomic and functional content of complex microbial communities by metagenomic sequencing on short-read NGS platforms. Remarkably, we find that the alignment-identity threshold range of the vast majority of hits of type 1 peptides from the current data set of 1,496,257 million proteins completely overlaps with the threshold ranges observed historically from much smaller data sets. The immediate application to metagenomics, of course, is that short peptides translated from short NGS reads are actually long enough to carry structural signatures causing them to align to their structural homologs.

### Applications to protein discovery

The tendency of short protein fragments to align to their structural homologs is a confounding factor in functional annotation due to the multiplicity of functions of homologous protein domains (same domain may perform multiple biochemical functions). However, the strong bias of type 1 peptide alignments to parental and non-parental KO-families that share similar EC number profiles leads to an intriguing idea that small peptides (originating from full length proteins) could be used as markers for predicting protein function or perhaps EC number profiles. In such cases, proteins having limited “global sequence similarity” with well characterized proteins (e.g. hard targets [36]) may nonetheless carry small peptides which can achieve significant alignments with these peptide markers. The most likely candidates for these proteins would be those whose evolutionary constraints are fundamentally different than what is observed in the known space (e.g. substitution patterns not evenly distributed but still somehow constrained).

### Applications to microbiome-wide and genome-wide association studies

To accommodate the alignment behaviors of type 1 peptides, we weighted significant hits by the frequency of hits to allow higher precision measurements of those peptides for which high-probability assignments could be made. A second improvement in assignment accuracy was to threshold peptides with alignment behaviors resembling type 2 peptides from the “unknown” protein space (e.g. low hit frequency and low percent identity). Together, these strategies improved quantitative estimates from type 3 data sets by a factor of 10 (see Methods for details). Because these “hard proteins” constitute significant proportions of known genomes (averaging 30 % of genomic content) and typically make up peptides that are predicted from 30 to 70 % of metagenomic reads, the improvements made by our criteria will have a dramatic effect on metagenomics applications where highly accurate, quantitative measurements of taxa and protein functional categories drive success of the experiments. For example, MWAS and GWAS experiments focused on microbiome traits depend exclusively on accurate measurements [37, 38] to limit type I and type II errors. The inherent biological noise, combined with sample error in these experiments, requires significant biological replication to appropriately power such experiments, and even when appropriately powered, false discovery rates still remain relatively high [39].

It should be noted that even with our thresholding designed from type 2 peptide behaviors, roughly 10 % of the proteins from the “hard protein” space were removed from the set of reference proteins for generating our type 2 peptide data sets because they displayed alignment behavior (70 % alignment length and 70 % identity) bordering type 1 peptides. These proteins are likely to occur at similar proportions in most metagenomic data sets. It seems likely that these proteins represent remote homologues of proteins in our database. They will remain a challenge for further improvement and refinement of data filtering and processing. Of the remaining hard proteins, >95 % of the peptides could be easily filtered using our criteria (low hit frequency and low percent identity). As new protein families are discovered, the alignment landscape will continue to expand, moving more type 2 peptides into the type 1 category and expanding the continuous landscape of the “known” protein universe. However, it is clear that the gap between the known and unknown portions of the universe is not likely to narrow substantially in the near future. Until then, removing the effects of alignments from peptides in this category has the huge advantage of improving the quantitative accuracy of measuring functions in the “known” universe, and that alone is cause for implementation.

## Conclusions

Our detailed analysis of short peptides shows that their alignment behavior is non-random and driven by structural properties. Although alignment patterns are constrained to structurally-related folds, these folds have been dispersed across proteins with a variety of functions by evolutionary processes, impairing accurate functional assignment even when peptides originate from well-defined proteins. The error-prone nature of functional assignments can, however, be minimized by weighting abundance predictions by the frequency of significant hits. In contrast to known protein families, peptides from hypothetical proteins have very distinct alignment patterns, allowing them to be easily filtered. By filtering out peptides originating from the “unknown protein space” and then appropriately weighting the contributions of remaining peptides, quantification of peptide distributions are much more accurate and will improve quantitative estimates of functions from metagenomic data.

## Methods

### Reference protein dataset

A list of all “bacterial KEGG entries” was obtained from the KEGG website (a total of 2910 entries) and corresponding protein sequences along with their annotations (functions, pathways and KO-families) were fetched from the M5nr [27] database. It yielded total 1, 496, 257 protein sequences covering 6327 unique KO-families.

### Datasets to study the alignment behavior of short peptides (Test case type1)

To study the alignment behavior of short peptides, we randomly picked a member protein from each KO-family present in our “Reference set” (total 6327 members were picked), and used it to generate, three equal-length and non-identical peptides (total 18,981 peptides/KO-family). To represent the multiple lengths of NGS reads (33–250 bp) we generated eight such test cases where length of peptides ranged from 11 to 81 (Test case Type1_11aa to Type1_81aa). This set is designed to represents the behavior of peptides originating from the “known” protein universe.

### Dataset to evaluate the impact of reads from uncharacterized genes (Test case type2)

To emulate the presence of reads originating from uncharacterized genes, we selected around 1.1 million hypothetical proteins (computationally predicted as proteins) with no membership to any KO-family i.e. biological roles of these proteins are not known (data source is M5nr database). To remove the redundancy of the data and computational burden, proteins were clustered with 70 % identity cutoff (using CD-hit [40]), which yielded 769,053 clusters. Finally we aligned representative member of each cluster against the “Reference set” (using BLASTP), to discard those that were homologous to “Reference set” proteins and therefore may share similar fold and function.

Since the quality of homology based fold prediction is proportional to the degree of sequence identity and alignment coverage between template and the target proteins [41], we generated three different test cases based on the alignment criteria to define the homology. For the first set (Type2.1), representative hypothetical proteins finding homologous counterparts (global alignment coverage of query protein > =70 % and alignment-identity > =70 %) within “Reference set” were removed since these alignment criteria are good enough to assign structural fold and/or protein family to an unknown protein, therefore such proteins do not fit the criteria of uncharacterized protein. For the second (Type2.2) and third (Type2.3) sets alignment identity criteria was relaxed to 50 and 35 % respectively [41]. Total of 6741 proteins were removed in the first set (762,312 remaining), whereas this number is 18,694 and 96,958 for second and third set (750,359 & 672,095 remaining) respectively. Each of the remaining proteins, within each test set, were used to generate three constant length (31aa long), non-identical peptides.

Peptides from our first, second and third sets (total 2,279,736, 2,244,375 and 2,010,159 peptides respectively) were aligned to “Reference set” proteins and their alignment behavior is detailed in the results section. Our approach was based on the premise that filtering uncharacterized proteins on the basis of their global sequence similarities with the “Reference set” proteins does not reduce their probability of having small/local alignments with “Reference set” proteins. Such hits eventually can influence the abundance profile calculations.

### Large simulated set to test ‘Frequency weighted read count’ method (Test case type3)

To evaluate our ‘Frequency weighted read count’ protocol, we generated around 5 million peptides covering all 6327 KO-families of our “Reference set”. As first step 10 % members of each KO-family were selected (i.e. 20 members from a KO-family with 200 members) and that resulted into total 180,510 proteins. From each of these selected proteins we randomly generated 20–40 equal-length (31amino-acids) and non-identical peptides, which yielded total 5,412,049 peptides representing real protein fragments with known KO-families assignments.

To study their alignment behavior, these (5,412,049) peptides were aligned against “Reference set” proteins (used BLASTP with default parameters). It had generated around a hundred million (112,3871715) hits, averaging 207.66 hits per peptide.

### Statistical analysis

Alignment behavior of blast-hits of peptide to parental and non-parental proteins overlaps significantly irrespective of the length of the peptides (Test case type1_11-81aa, Additional file 1: Figure S1-S8). For statistical verification of this phenomenon we performed one way ANOVA over differences of alignment identity of random 500 hits to parent and non-parent KO-families and its repeated 100 and finally average p-value is calculated.

To quantify the effectiveness of “filter enabled frequency-weighted method”, the extent of abundance profile correction was calculated by averaging the absolute differences of calculated read count vs real read count of all the KO-families. While mean degree of deviation for ‘Selected_4K_KO_Hits’ test case was 9.49 (SD 16.38), the same for its composite with ‘type 2.3’ is 114.57 (SD 199.85, ‘no filtering of unknown peptides’) and 23.6 (SD 36.6, ‘filter applied’). Therefore we see around 11 fold (114.57/9.49) degree of deviation from the original read count when peptides from hypothetical proteins are introduced and around 10 fold (114.5–23.6/9.4) correction when the filter-enabled “frequency weighted read count method” was applied.

Receiver operating characteristic (ROC) curves were generated to evaluate the ability of multiple combinations of parameters such as alignment coverage and identity to assign true KOs to short peptides. Three different ROC curves based on peptide lengths (31, 51 and 71aa) were generated. For each individual plot, true-positive and false-positive rates for combinations of alignment-coverages (ranging 50–80 % with increments of 10 %) and alignment-identities (ranging 40–90 % with increments of 10 %) were plotted. We picked minimum coverage and identity values (50 and 40 % respectively) which are significant enough to establish an evolutionary relationship between two protein sequences. For each parameter combinations, 10,000 alignments of Type-1 short peptides were used to calculate the true-positive (TP /TP + FN) and false-positive (FP/FP + TN) rates.

## Availability of supporting data

All the test-cases, alignment output and python scripts used in this study are hosted at (http://cage.unl.edu/DataPeptide). A “supplementary information” file carrying Additional file 1: Figure S1–44 and data description is also provided along with the main manuscript.

## Additional file

Additional file 1:
**An additional file is available along with the online version of this paper.** Additional file 1 not only contains the Figures S1–45 but also contains a detailed description of the nature of files and data shared on (http://cage.unl.edu/DataPeptide). (DOCX 14611 kb)

## Abbreviations

EC: enzyme commission
FN: total falls negative calls
FP: total false positive calls
GWAS: genome-wide association studies
KEGG: kyoto encyclopedia of genes and genomes
KO: KEGG-orthology group
MWAS: metabolome-wide association studies
NGS: next-generation sequencing
SD: standard deviation
SSM: sequence similarity measures (percent identity, e-value, alignment coverage)
TN: total true negative calls
TP: total true positive calls

## References

[1] Schuster SC (2007). Next-generation sequencing transforms today’s biology. Nat Methods.

[2] Tyson GW, Chapman J, Hugenholtz P, Allen EE, Ram RJ, Richardson PM (2004). Community structure and metabolism through reconstruction of microbial genomes from the environment. Nature.

[3] Venter JC, Remington K, Heidelberg JF, Halpern AL, Rusch D, Eisen JA (2004). Environmental genome shotgun sequencing of the Sargasso Sea. Science.

[4] Edwards RA, Rodriguez-Brito B, Wegley L, Haynes M, Breitbart M, Peterson DM (2006). Using pyrosequencing to shed light on deep mine microbial ecology. BMC Genomics.

[5] Gill SR, Pop M, Deboy RT, Eckburg PB, Turnbaugh PJ, Samuel BS (2006). Metagenomic analysis of the human distal gut microbiome. Science.

[6] National Research Council (US) Committee on Metagenomics (2007). Challenges and functional applications, the new science of metagenomics: revealing the secrets of our microbial planet.

[7] Turnbaugh PJ, Hamady M, Yatsunenko T, Cantarel BL, Duncan A, Ley RE (2009). A core gut microbiome in obese and lean twins. Nature.

[8] Qin J, Li R, Raes J, Arumugam M, Burgdorf KS, Manichanh C (2010). A human gut microbial gene catalogue established by metagenomic sequencing. Nature.

[9] Wilson CA, Kreychman J, Gerstein M (2000). Assessing annotation transfer for genomics: quantifying the relations between protein sequence, structure and function through traditional and probabilistic scores. J Mol Biol.

[10] Roy A, Kucukural A, Zhang Y (2010). I-TASSER: a unified platform for automated protein structure and function prediction. Nat Protoc.

[11] Roy A, Yang J, Zhang Y (2012). COFACTOR: an accurate comparative algorithm for structure-based protein function annotation. Nucleic Acids Res.

[12] Todd AE, Orengo CA, Thornton JM (2001). Evolution of function in protein superfamilies, from a structural perspective. J Mol Biol.

[13] Omelchenko MV, Galperin MY, Wolf YI, Koonin EV (2010). Non-homologous isofunctional enzymes: a systematic analysis of alternative solutions in enzyme evolution. Biol Direct.

[14] Ekman D, Björklund AK, Frey-Skött J, Elofsson A (2005). Multi-domain proteins in the three kingdoms of life: orphan domains and other unassigned regions. J Mol Biol.

[15] Wang M, Kurland CG, Caetano-Anollés G (2011). Reductive evolution of proteomes and protein structures. Proc Natl Acad Sci U S A.

[16] Huson DH, Auch AF, Qi J, Schuster SC (2007). MEGAN analysis of metagenomic data. Genome Res.

[17] Meyer F, Paarmann D, D’Souza M, Olson R, Glass EM, Kubal M (2008). The metagenomics RAST server - a public resource for the automatic phylogenetic and functional analysis of metagenomes. BMC Bioinformatics.

[18] Abubucker S, Segata N, Goll J, Schubert AM, Izard J, Cantarel BL (2012). Metabolic reconstruction for metagenomic data and its application to the human microbiome. PLoS Comput Biol.

[19] Huson DH, Xie C (2014). A poor man’s BLASTX--high-throughput metagenomic protein database search using PAUDA. Bioinforma Oxf Engl.

[20] Bono H, Ogata H, Goto S, Kanehisa M (1998). Reconstruction of amino acid biosynthesis pathways from the complete genome sequence. Genome Res.

[21] Bragina A, Oberauner-Wappis L, Zachow C, Halwachs B, Thallinger GG, Müller H (2014). The Sphagnum microbiome supports bog ecosystem functioning under extreme conditions. Mol Ecol.

[22] Gupta SS, Mohammed MH, Ghosh TS, Kanungo S, Nair GB, Mande SS (2011). Metagenome of the gut of a malnourished child. Gut Pathog.

[23] Smedile F, Messina E, La Cono V, Tsoy O, Monticelli LS, Borghini M (2013). Metagenomic analysis of hadopelagic microbial assemblages thriving at the deepest part of Mediterranean Sea, Matapan-Vavilov Deep. Environ Microbiol.

[24] Moitinho-Silva L, Seridi L, Ryu T, Voolstra CR, Ravasi T, Hentschel U (2014). Revealing microbial functional activities in the Red Sea sponge Stylissa carteri by metatranscriptomics. Environ Microbiol.

[25] Xing M, Hou Z, Yuan J, Liu Y, Qu Y, Liu B (2013). Taxonomic and functional metagenomic profiling of gastrointestinal tract microbiome of the farmed adult turbot (Scophthalmus maximus). FEMS Microbiol Ecol.

[26] Overbeek R, Begley T, Butler RM, Choudhuri JV, Chuang H-Y, Cohoon M (2005). The Subsystems Approach to Genome Annotation and its Use in the Project to Annotate 1000 Genomes. Nucleic Acids Res.

[27] Wilke A, Harrison T, Wilkening J, Field D, Glass EM, Kyrpides N (2012). The M5nr: a novel non-redundant database containing protein sequences and annotations from multiple sources and associated tools. BMC Bioinformatics.

[28] Chothia C (1992). Proteins. One thousand families for the molecular biologist. Nature.

[29] Zhang Y, Hubner IA, Arakaki AK, Shakhnovich E, Skolnick J (2006). On the origin and highly likely completeness of single-domain protein structures. Proc Natl Acad Sci U S A.

[30] Cusack S, Yaremchuk A, Krikliviy I, Tukalo M (1998). tRNA(Pro) anticodon recognition by Thermus thermophilus prolyl-tRNA synthetase. Struct.

[31] Xu D, Zhang Y (2013). Ab Initio structure prediction for Escherichia coli: towards genome-wide protein structure modeling and fold assignment. Sci Rep.

[32] Lesk AM, Chothia C (1980). How different amino acid sequences determine similar protein structures: the structure and evolutionary dynamics of the globins. J Mol Biol.

[33] Chothia C, Lesk AM (1986). The relation between the divergence of sequence and structure in proteins. EMBO J.

[34] Sander C, Schneider R (1991). Database of homology-derived protein structures and the structural meaning of sequence alignment. Proteins.

[35] Rost B (1999). Twilight zone of protein sequence alignments. Protein Eng.

[36] Sitao Wu YZ (2009). Protein structure prediction.

[37] Benson AK, Kelly SA, Legge R, Ma F, Low SJ, Kim J (2010). Individuality in gut microbiota composition is a complex polygenic trait shaped by multiple environmental and host genetic factors. Proc Natl Acad Sci U S A.

[38] Qin J, Li Y, Cai Z, Li S, Zhu J, Zhang F (2012). A metagenome-wide association study of gut microbiota in type 2 diabetes. Nature.

[39] Leamy LJ, Kelly SA, Nietfeldt J, Legge RM, Ma F, Hua K (2014). Host genetics and diet, but not immunoglobulin A expression, converge to shape compositional features of the gut microbiome in an advanced intercross population of mice. Genome Biol.

[40] Li W, Godzik A (2006). Cd-hit: a fast program for clustering and comparing large sets of protein or nucleotide sequences. Bioinforma Oxf Engl.

[41] Baker D, Sali A (2001). Protein structure prediction and structural genomics. Science.

