# Supplementary material for: Alignment behaviors of short peptides provide a roadmap for functional profiling of metagenomic data
Source: BMC Genomics. 2015 Dec 21;16:1080. doi: 10.1186/s12864-015-2272-z (PMC4687345; doi:10.1186/s12864-015-2272-z)
Supplement: Additional file 1: — An additional file is available along with the online version of this paper. Additional file 1 not only contains the Figures S1–45 but also contains a detailed description of the nature of files and data shared on (http://cage.unl.edu/DataPeptide). (DOCX 14611 kb) [file 12864_2015_2272_MOESM1_ESM.docx]

**Alignment behavior of short peptides provide a roadmap for functional profiling of metagenomic data**

Rohita Sinha^1^, Jennifer Clarke^1,2,3^ , Andrew K. Benson^1^

^1^Department of Food Science and Technology, University of Nebraska, Lincoln, NE, 68583, USA ^2^Department of Statistics, University of Nebraska, Lincoln, NE, 68583, USA ^3^Computational Sciences Initiative, University of Nebraska, Lincoln, NE, 68583 USA

# Supplementary Figures:

| 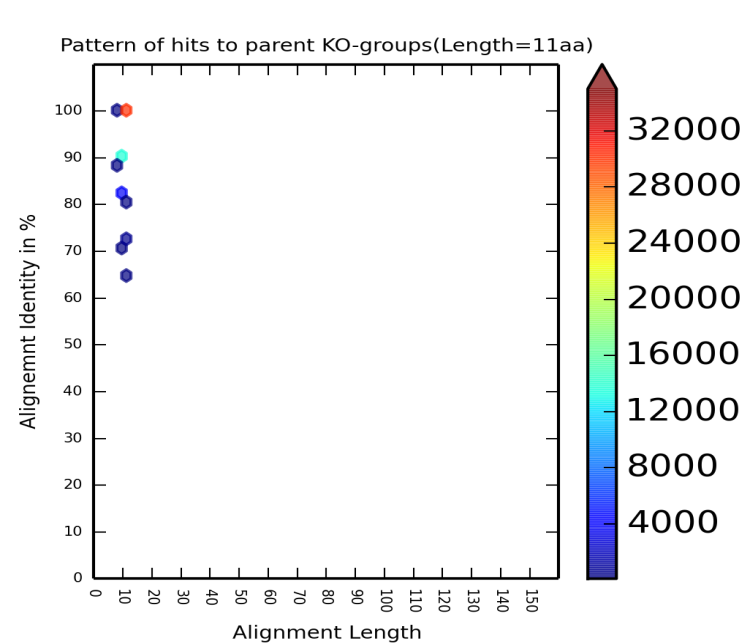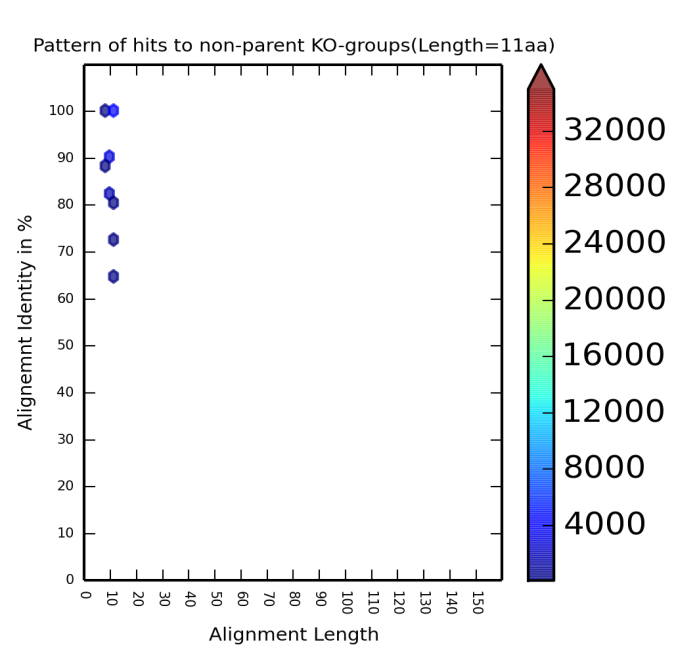  Figure S 1: Comparison of alignment behavior when the short peptides align to members of their parent KO-groups (Left panel) and when short peptides align to members of their non-parent KO-groups (Right panel). Length of peptides in the dataset is 11 amino acids. |
| --- |
| 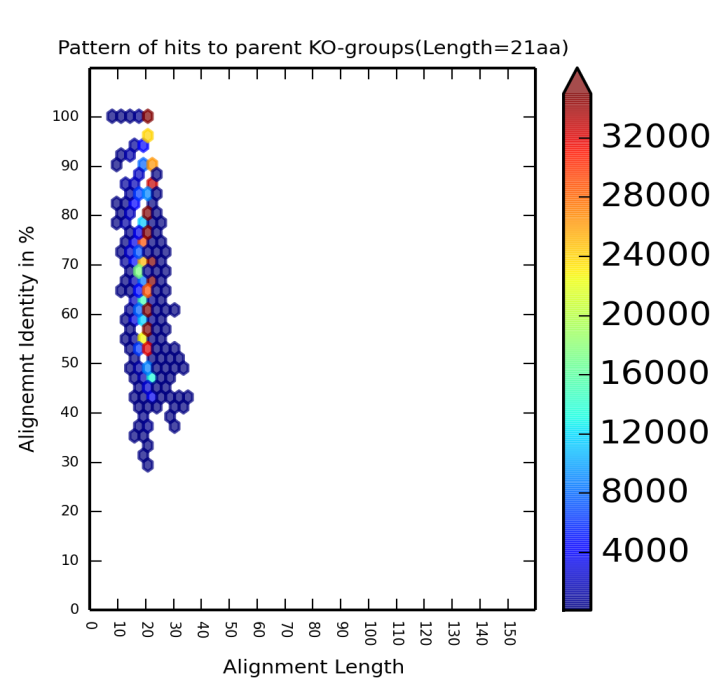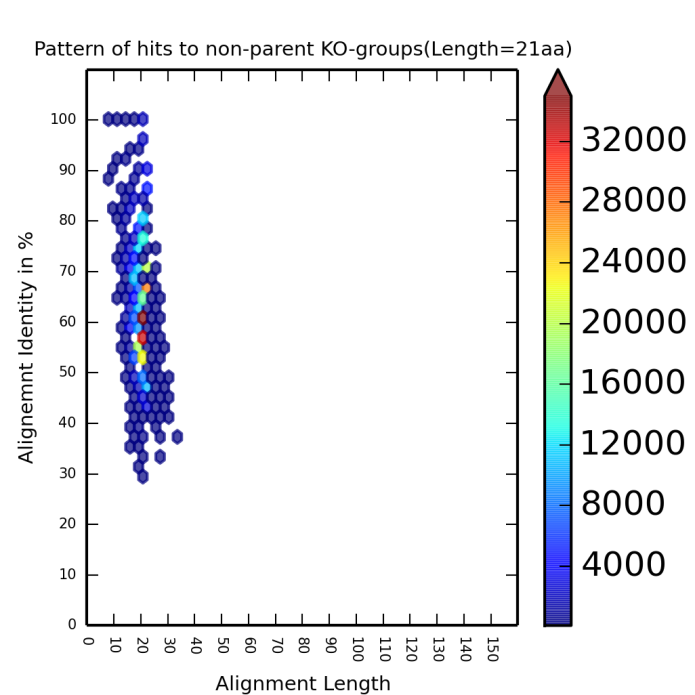  Figure S 2: Comparison of alignment behavior when the short peptides align to members of their parent KO-groups (Left panel) and when short peptides align to members of their non-parent KO-groups (Right panel). Length of peptides in the dataset is 21 amino acids. |
| 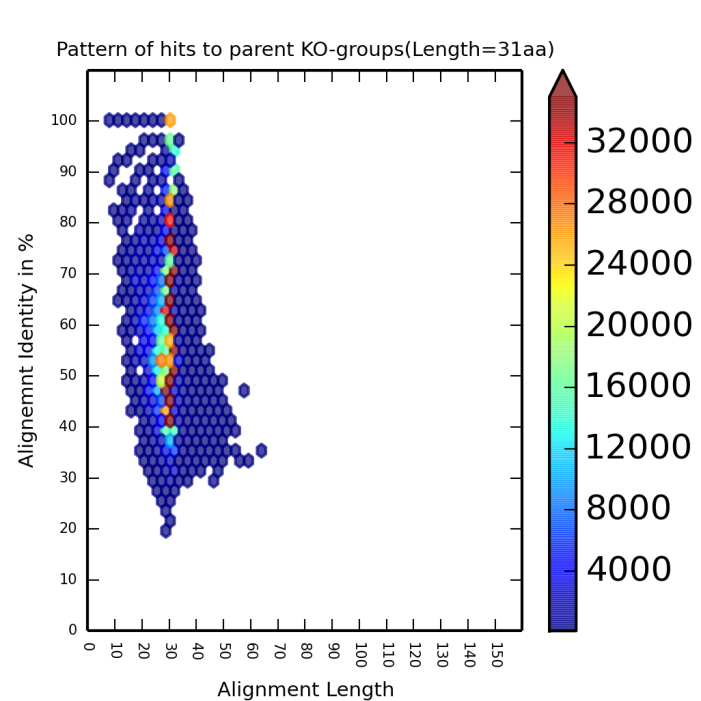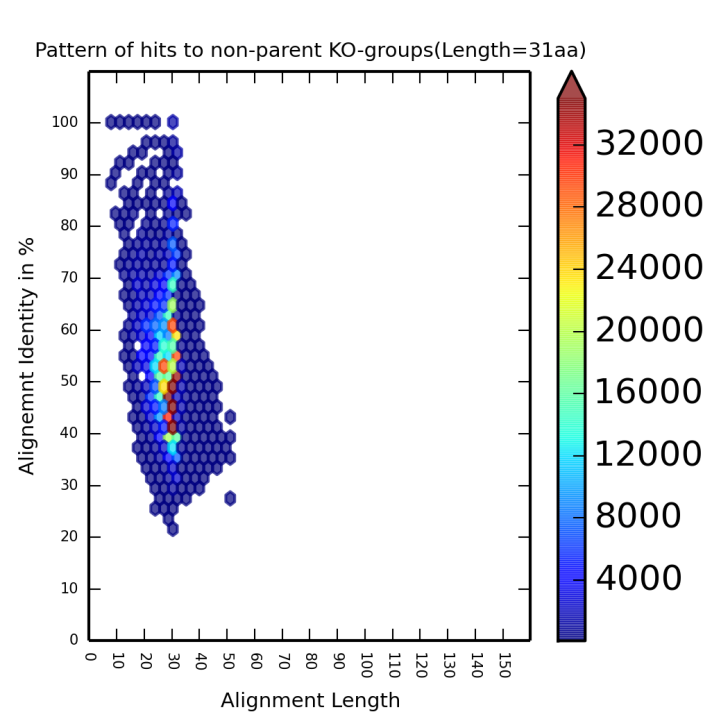  Figure S 3: Comparison of alignment behavior when the short peptides align to members of their parent KO-groups (Left panel) and when short peptides align to members of their non-parent KO-groups (Right panel). Length of peptides in the dataset is 31 amino acids |
| 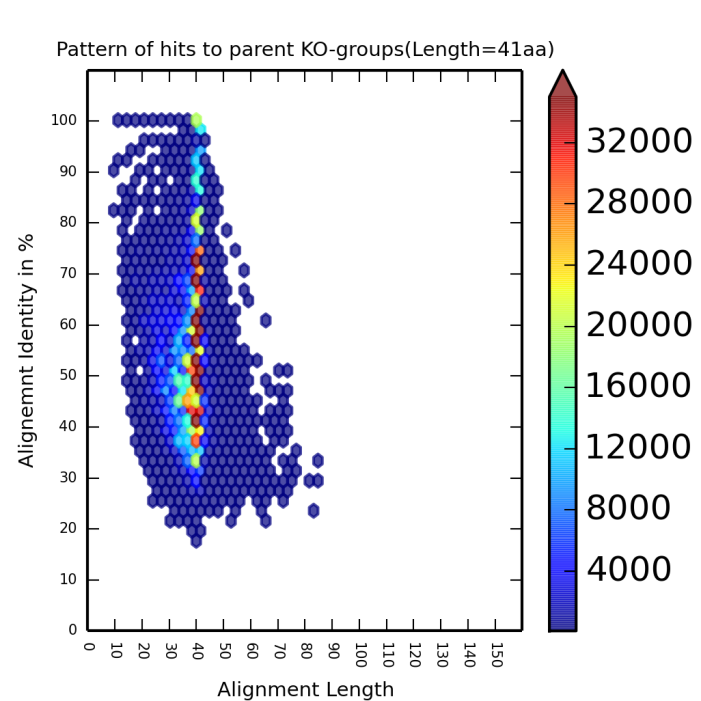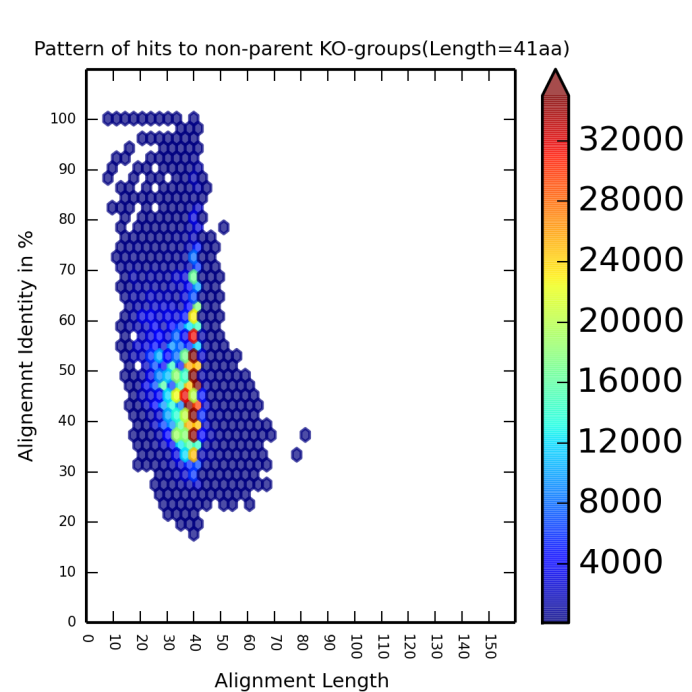  Figure S 4: Comparison of alignment behavior when the short peptides align to members of their parent KO-groups (Left panel) and when short peptides align to members of their non-parent KO-groups (Right panel). Length of peptides in the dataset is 41 amino acids. |
| 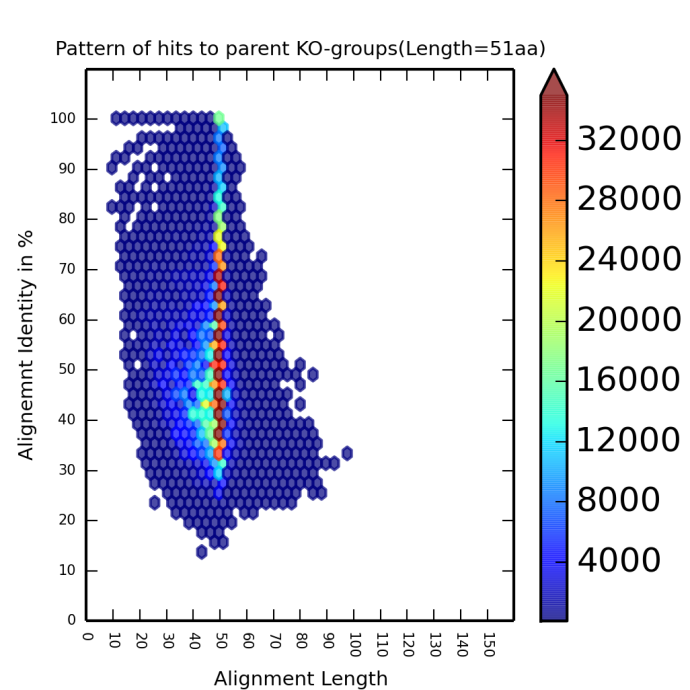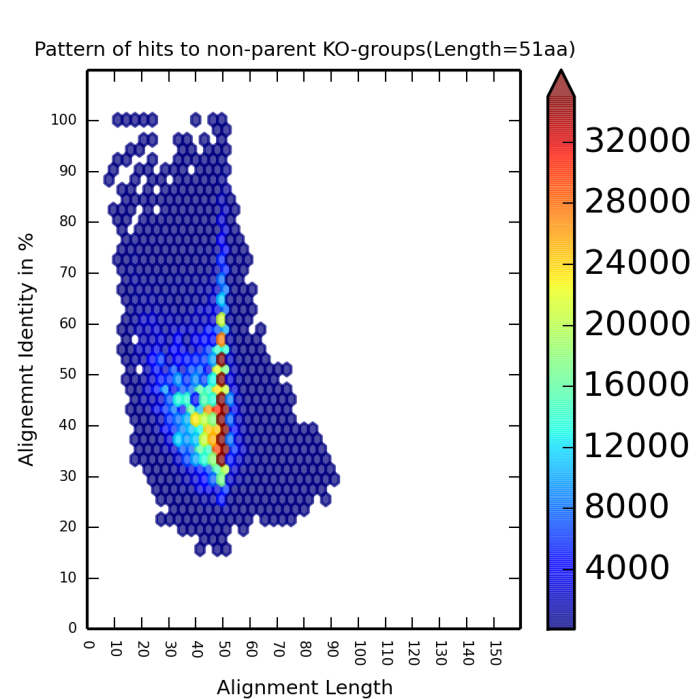  Figure S 5: Comparison of alignment behavior when the short peptides align to members of their parent KO-groups (Left panel) and when short peptides align to members of their non-parent KO-groups (Right panel). Length of peptides in the dataset is 51 amino acids. |
| 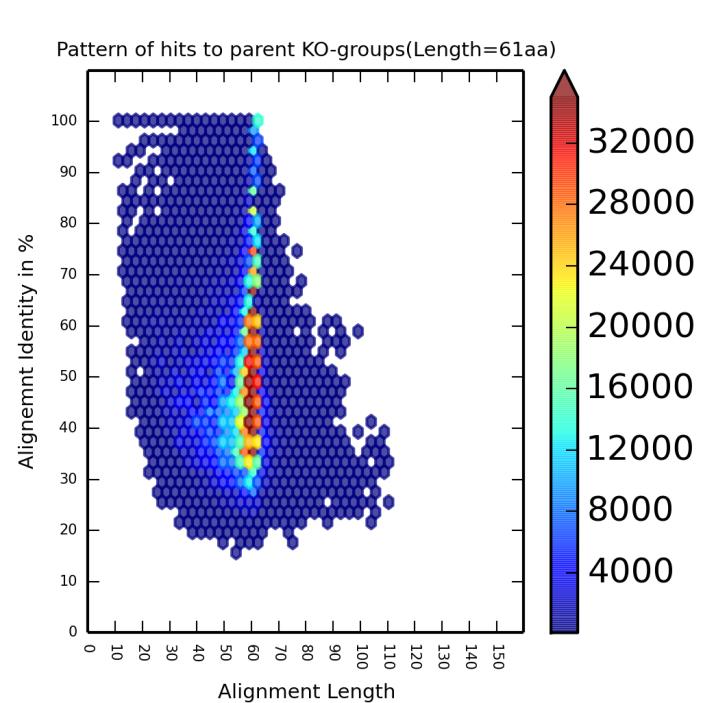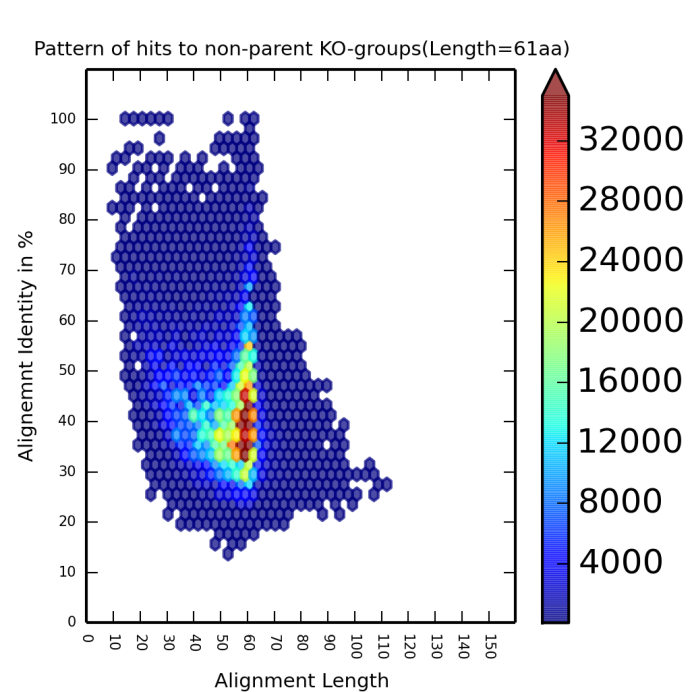  Figure S 6: Comparison of alignment behavior when the short peptides align to members of their parent KO-groups (Left panel) and when short peptides align to members of their non-parent KO-groups (Right panel). Length of peptides in the dataset is 61 amino acids |
| 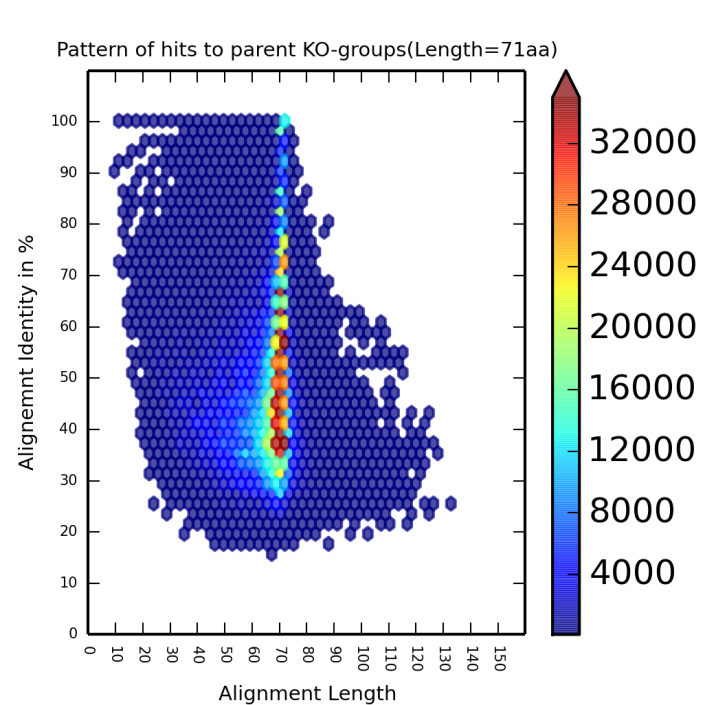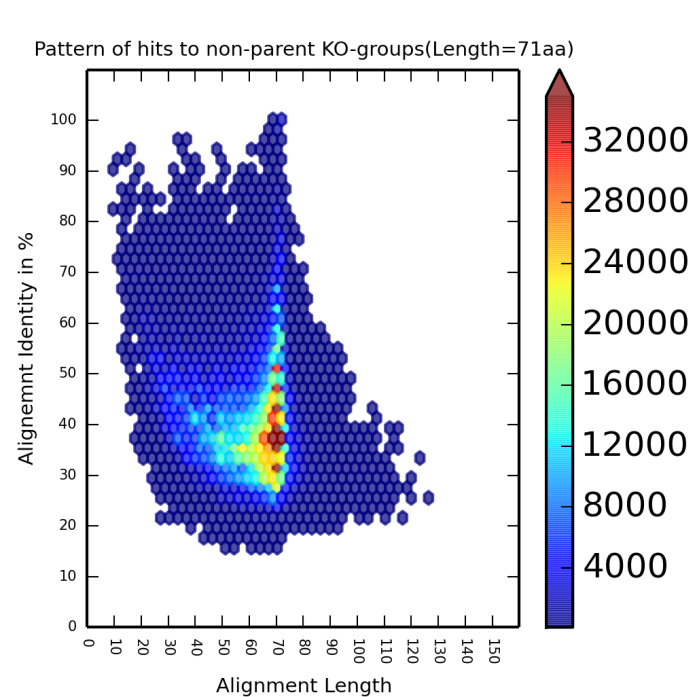  Figure S 7: Comparison of alignment behavior when the short peptides align to members of their parent KO-groups (Left panel) and when short peptides align to members of their non-parent KO-groups (Right panel). Length of peptides in the dataset is 71 amino acids |
| 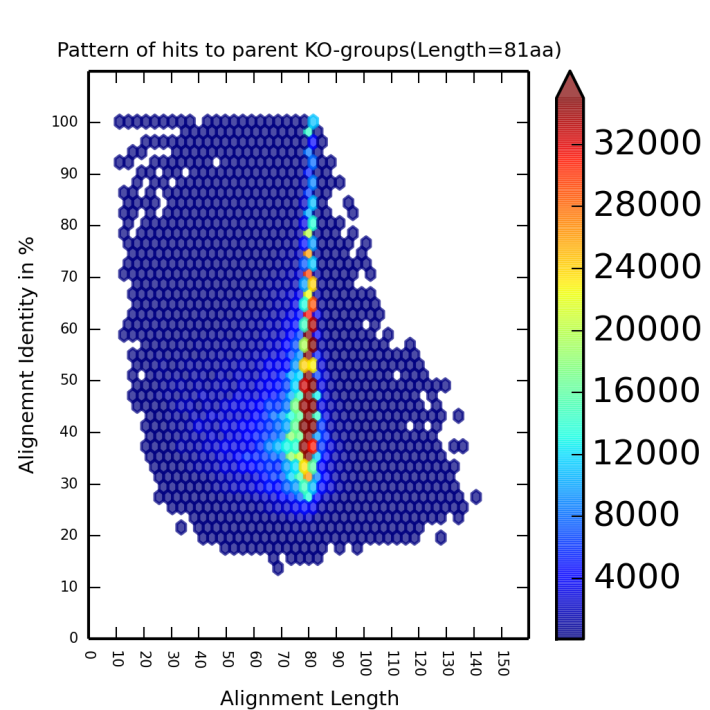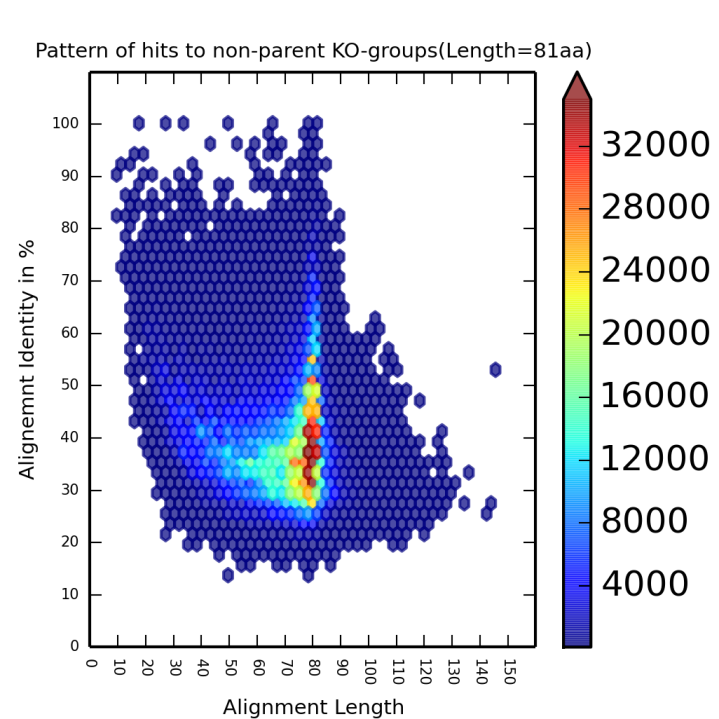  Figure S 8: Comparison of alignment behavior when the short peptides align to members of their parent KO-groups (Left panel) and when short peptides align to members of their non-parent KO-groups (Right panel). Length of peptides in the dataset is 81 amino acids |

| 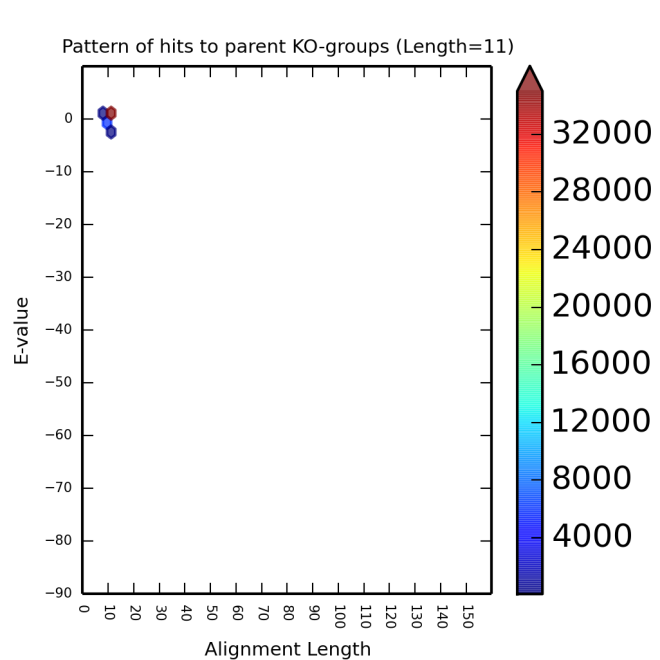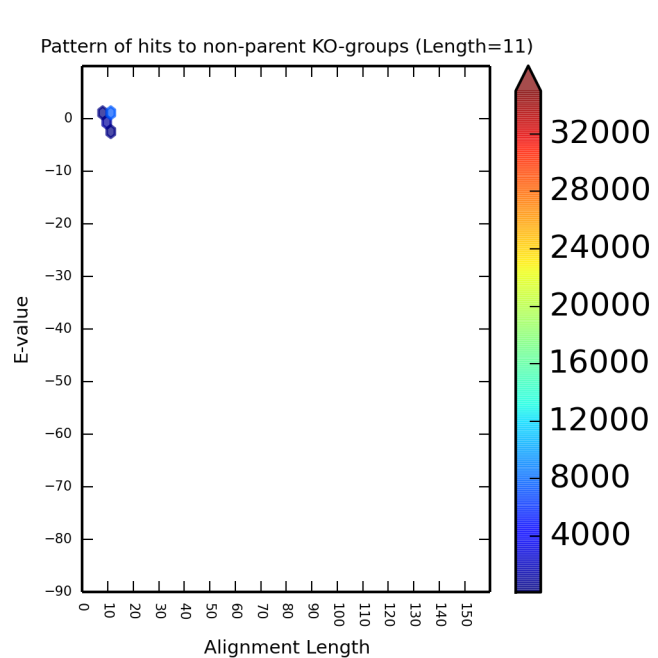  Figure S 9: Comparison of alignment behavior in terms of E-value and alignment length when the short peptides align to members of their parent KO-groups (Left panel) and when short peptides align to members of their non-parent KO-groups (Right panel). Length of peptides in the dataset is 11 amino acids. |
| --- |
| 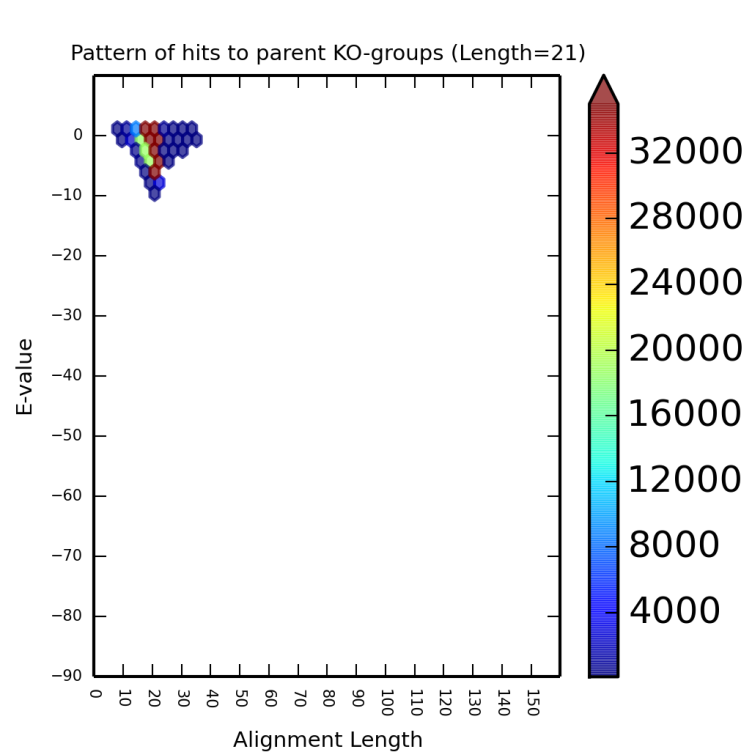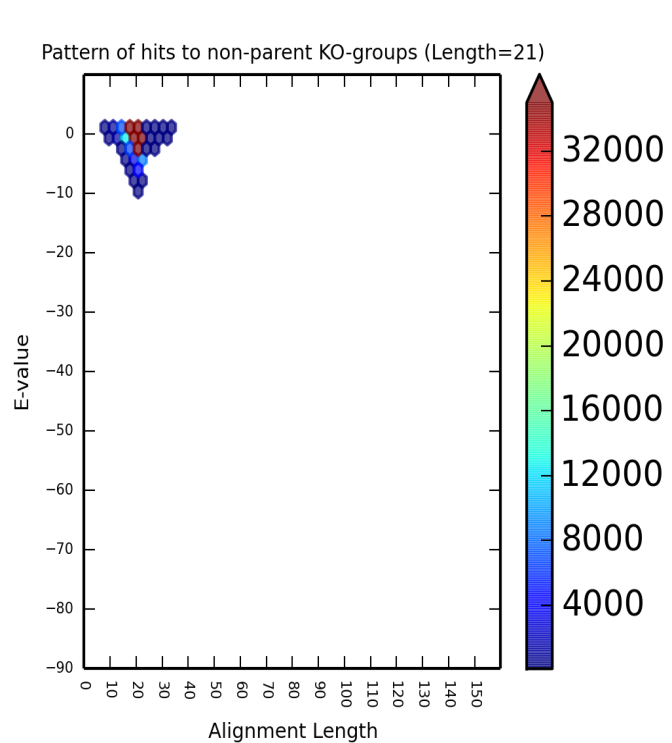  Figure S 10: Comparison of alignment behavior in terms of E-value and alignment length when the short peptides align to members of their parent KO-groups (Left panel) and when short peptides align to members of their non-parent KO-groups (Right panel). Length of peptides in the dataset is 21 amino acids. |
| 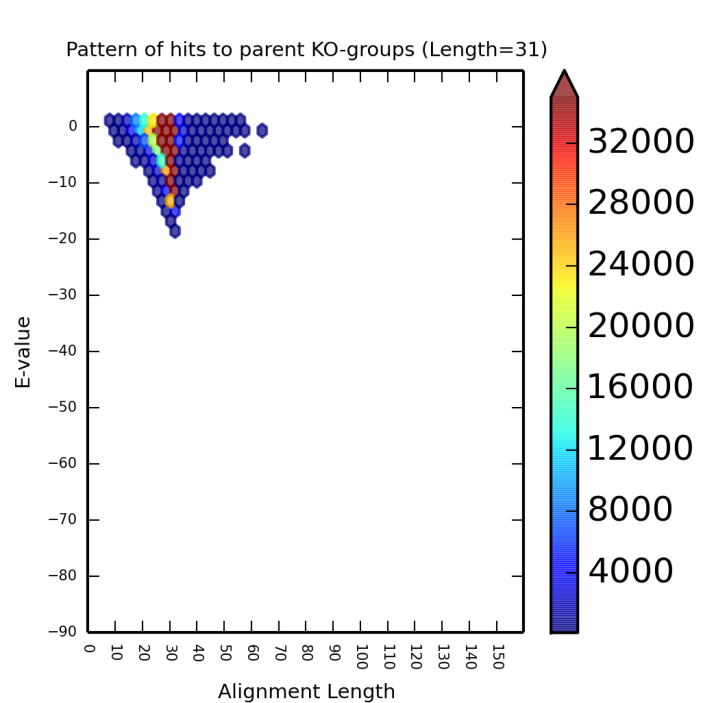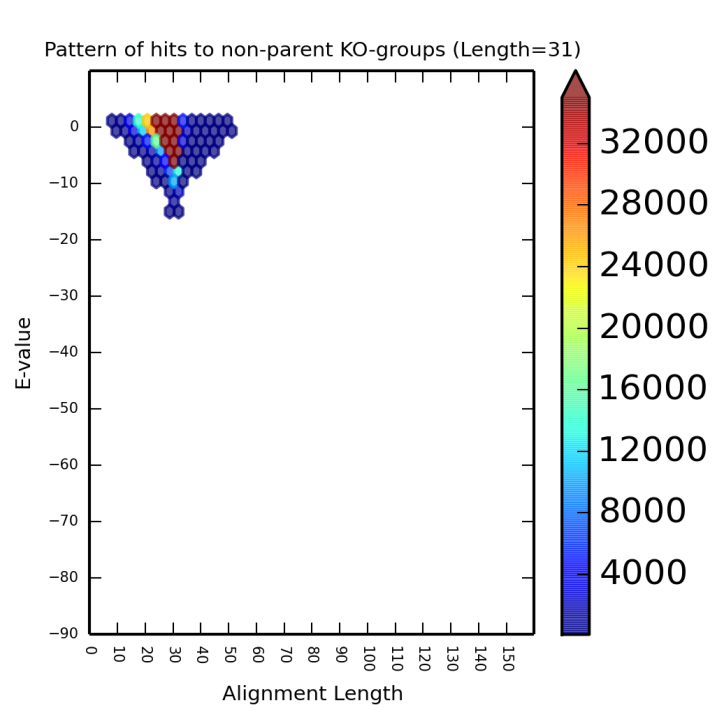  Figure S 11: Comparison of alignment behavior in terms of E-value and alignment length when the short peptides align to members of their parent KO-groups (Left panel) and when short peptides align to members of their non-parent KO-groups (Right panel). Length of peptides in the dataset is 31 amino acids. |
| 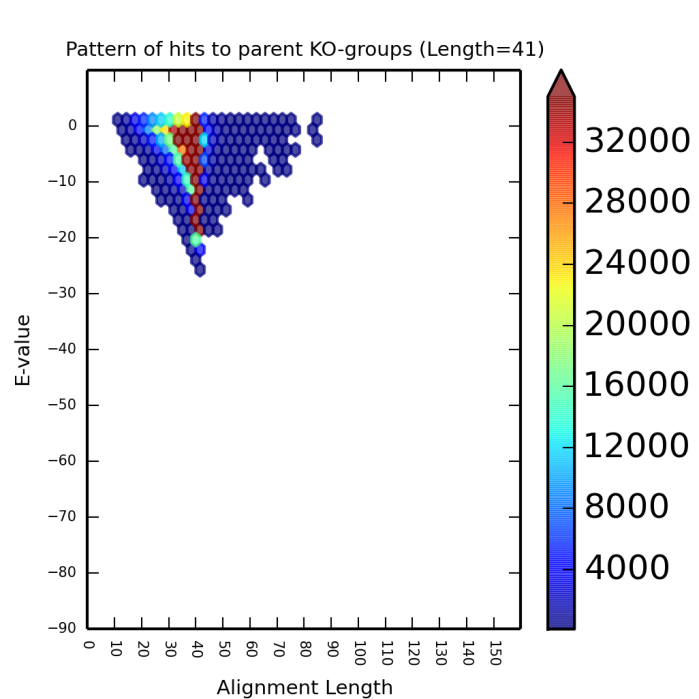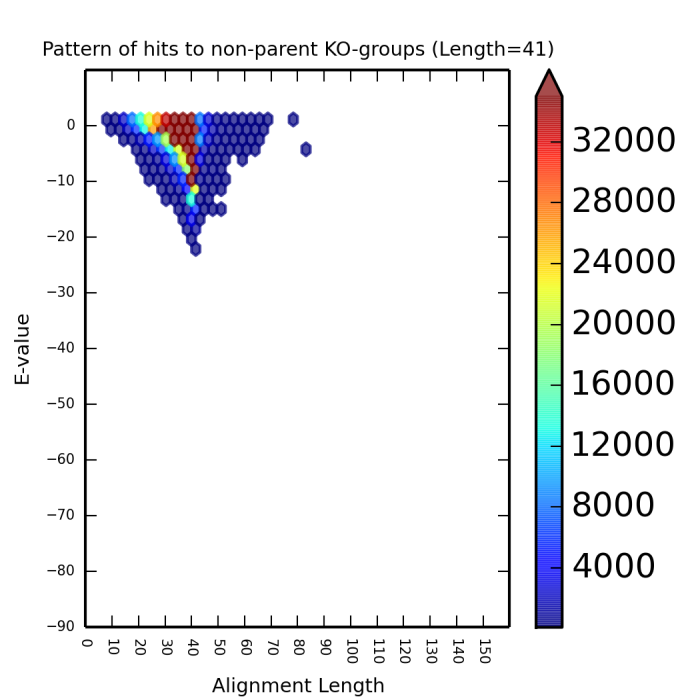  Figure S 12: Comparison of alignment behavior in terms of E-value and alignment length when the short peptides align to members of their parent KO-groups (Left panel) and when short peptides align to members of their non-parent KO-groups (Right panel). Length of peptides in the dataset is 41 amino acids. |
| 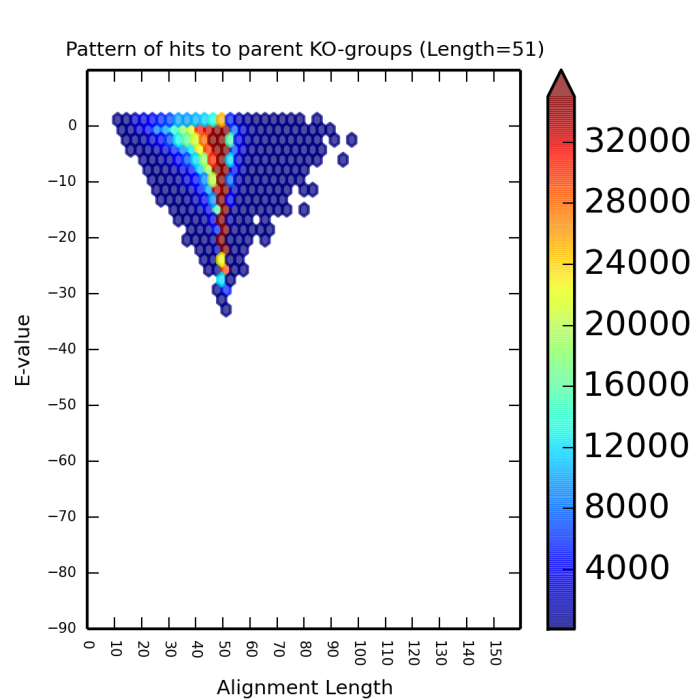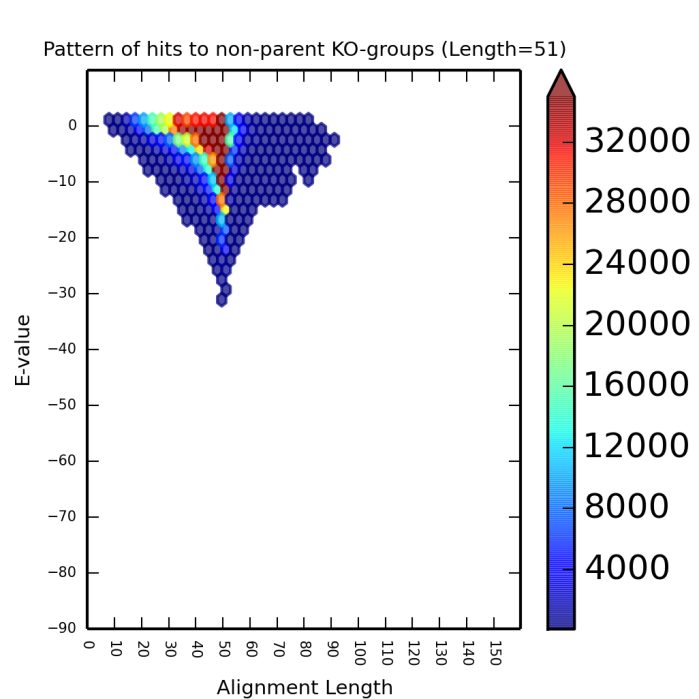  Figure S 13: Comparison of alignment behavior in terms of E-value and alignment length when the short peptides align to members of their parent KO-groups (Left panel) and when short peptides align to members of their non-parent KO-groups (Right panel). Length of peptides in the dataset is 51 amino acids. |
| 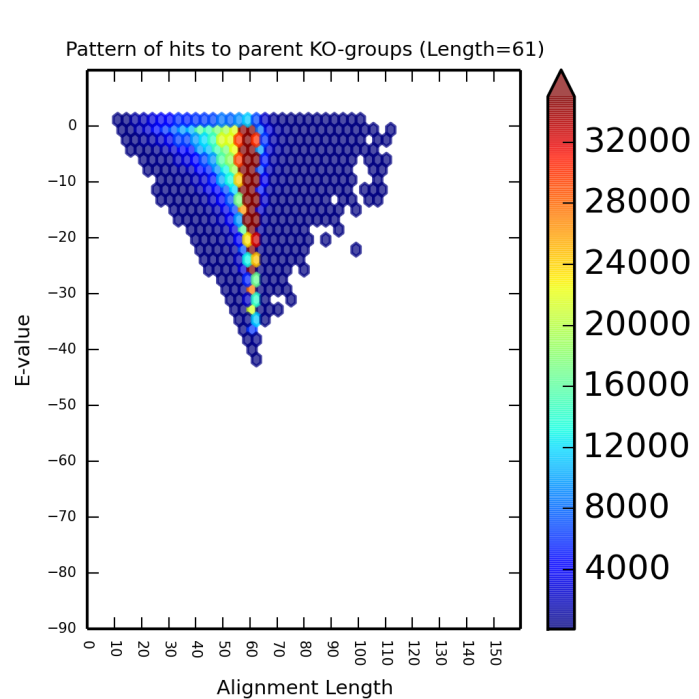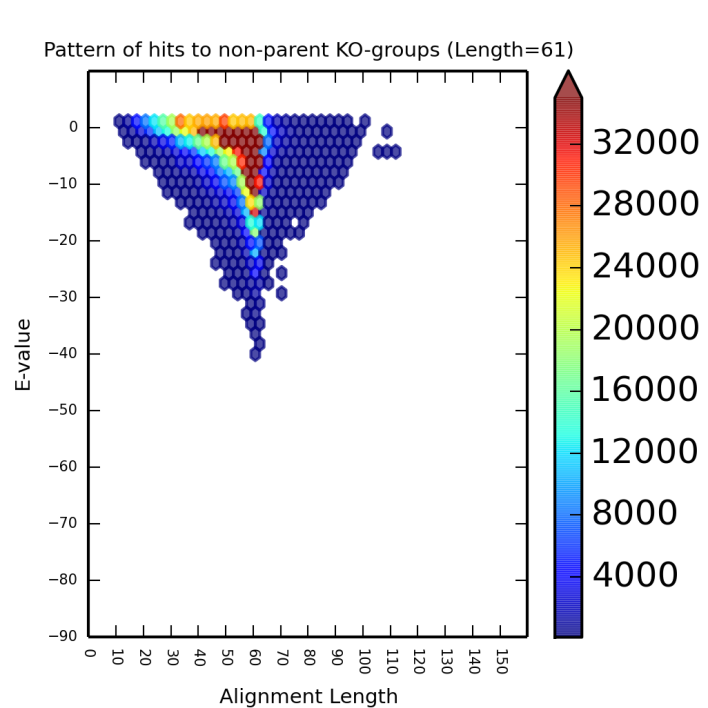  Figure S 14: Comparison of alignment behavior in terms of E-value and alignment length when the short peptides align to members of their parent KO-groups (Left panel) and when short peptides align to members of their non-parent KO-groups (Right panel). Length of peptides in the dataset is 61 amino acids. |
| 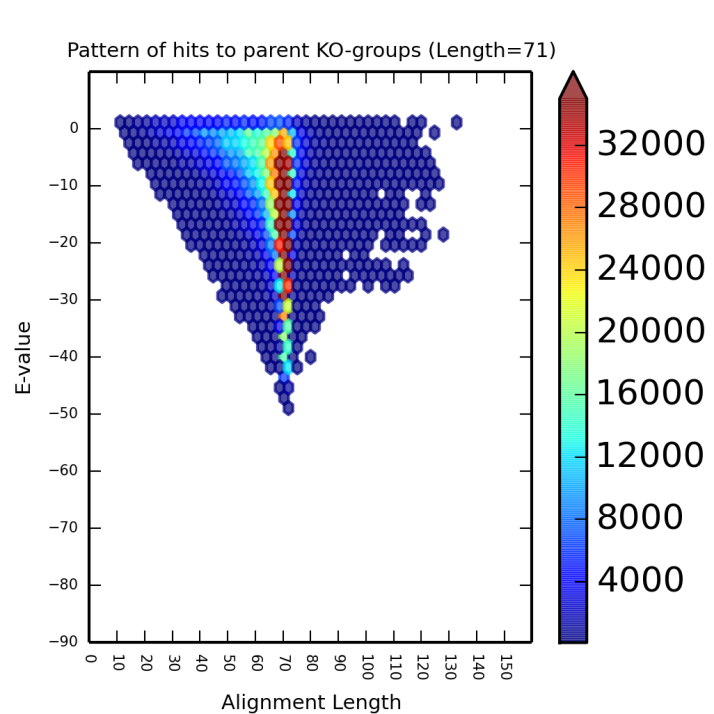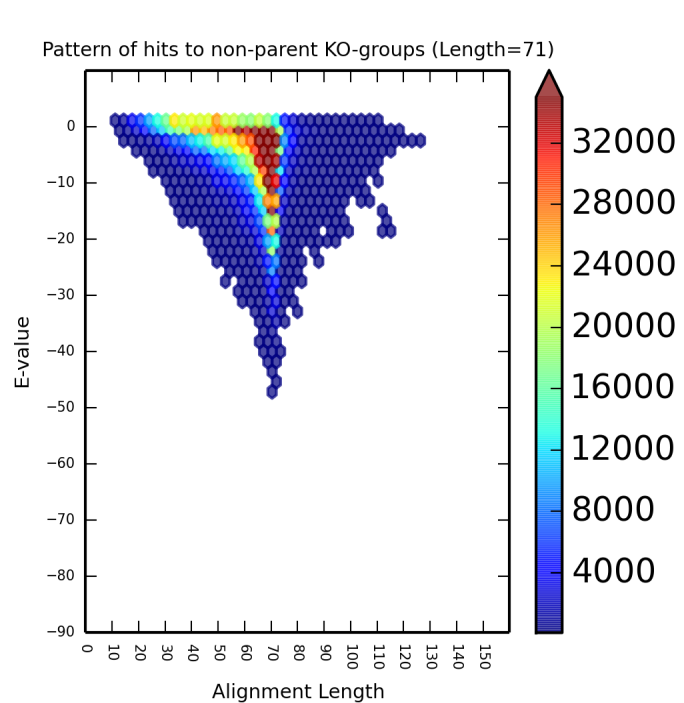  Figure S 15: Comparison of alignment behavior in terms of E-value and alignment length when the short peptides align to members of their parent KO-groups (Left panel) and when short peptides align to members of their non-parent KO-groups (Right panel). Length of peptides in the dataset is 71 amino acids. |
| 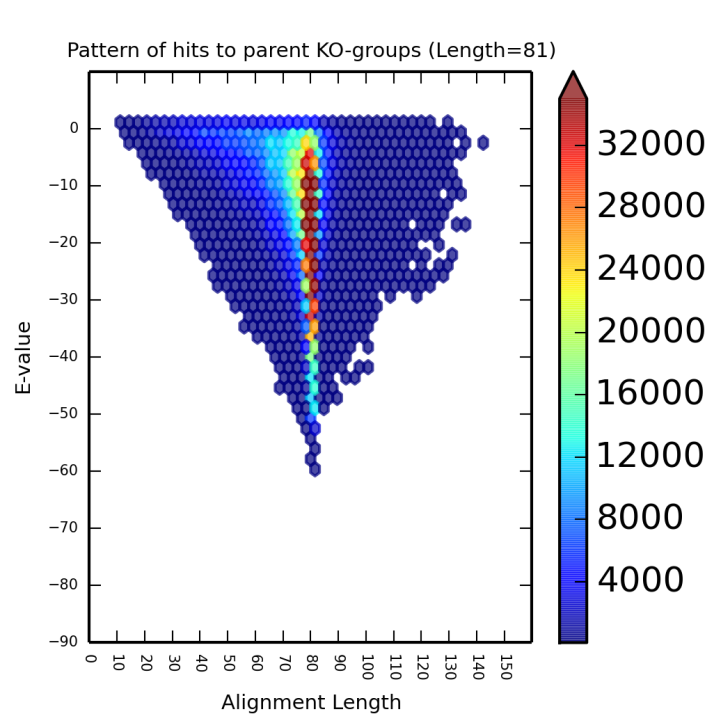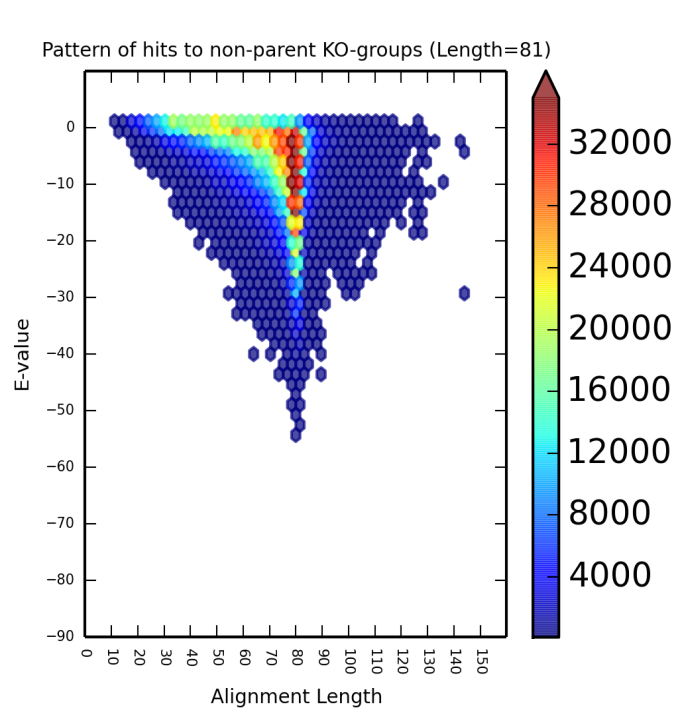  Figure S 16: Comparison of alignment behavior in terms of E-value and alignment length when the short peptides align to members of their parent KO-groups (Left panel) and when short peptides align to members of their non-parent KO-groups (Right panel). Length of peptides in the dataset is 81 amino acids. |

| 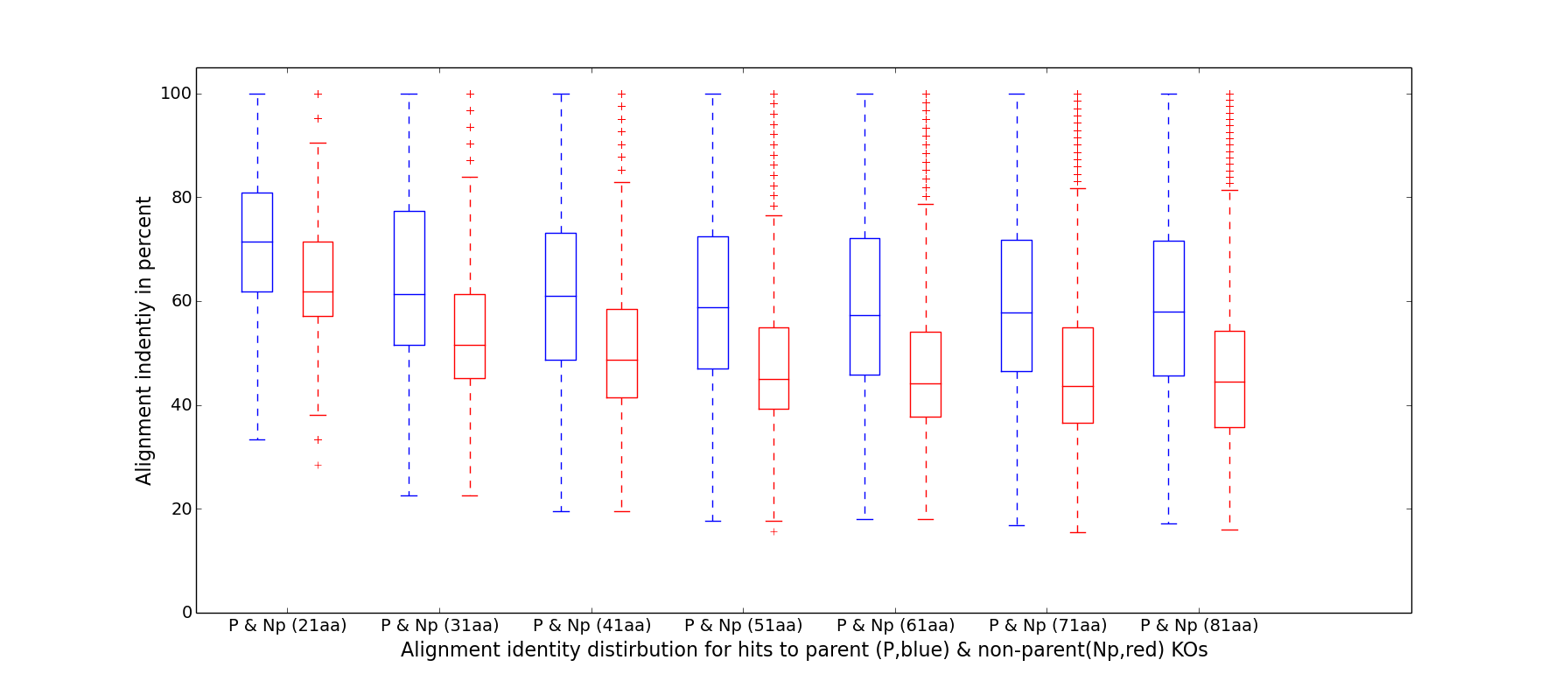  Figure S 17: Alignment identity distribution profiles for each peptide length (21-81aa), showing that it always ranges higher for hits to parent protein KO groups (Blue boxes) as compared to hits to non-parent KO groups (Red boxes). |
| --- |

| 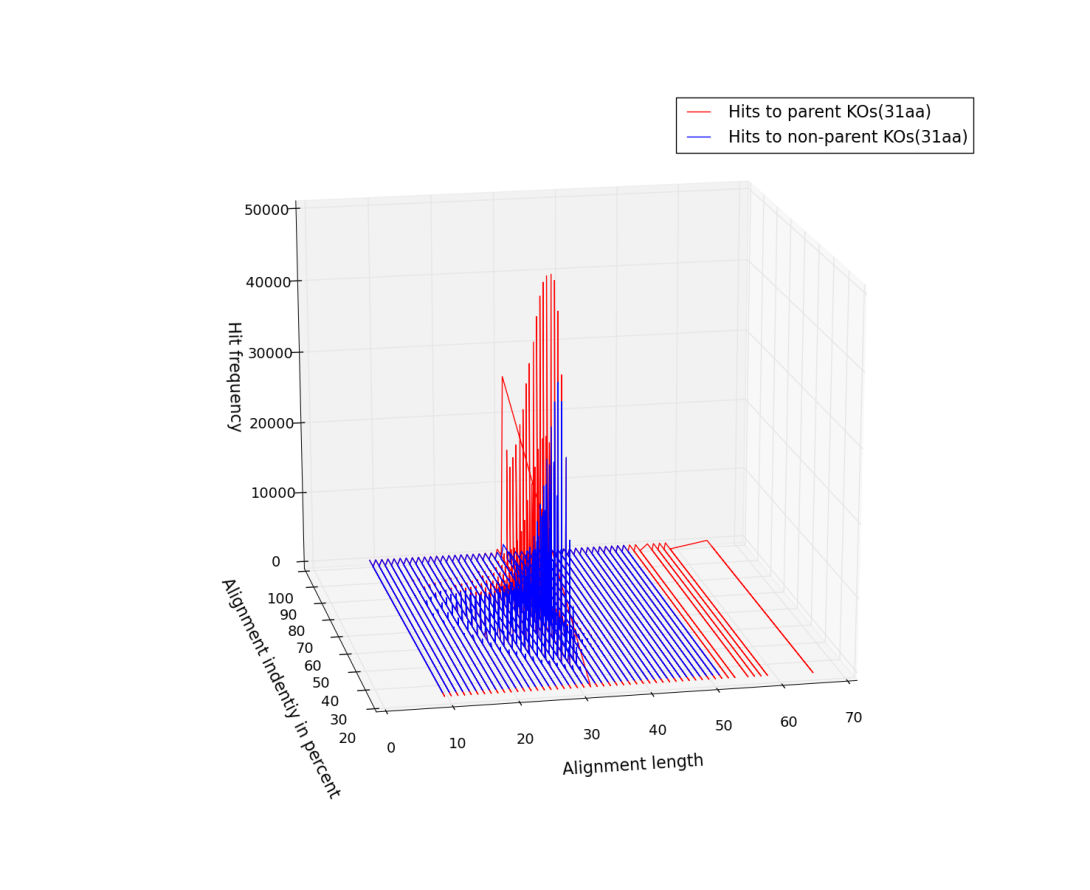  Figure S 18:3D plot of alignment length, alignment-identity and frequency of hits to parent KO groups (Red) and non-parent KO groups (Blue). Length of peptides in the dataset is 31 amino acids. Significant overlap of blue and red graphs substantiates the absence of sequence similarity cutoffs to distinguish these two kinds of alignments. |
| --- |

| 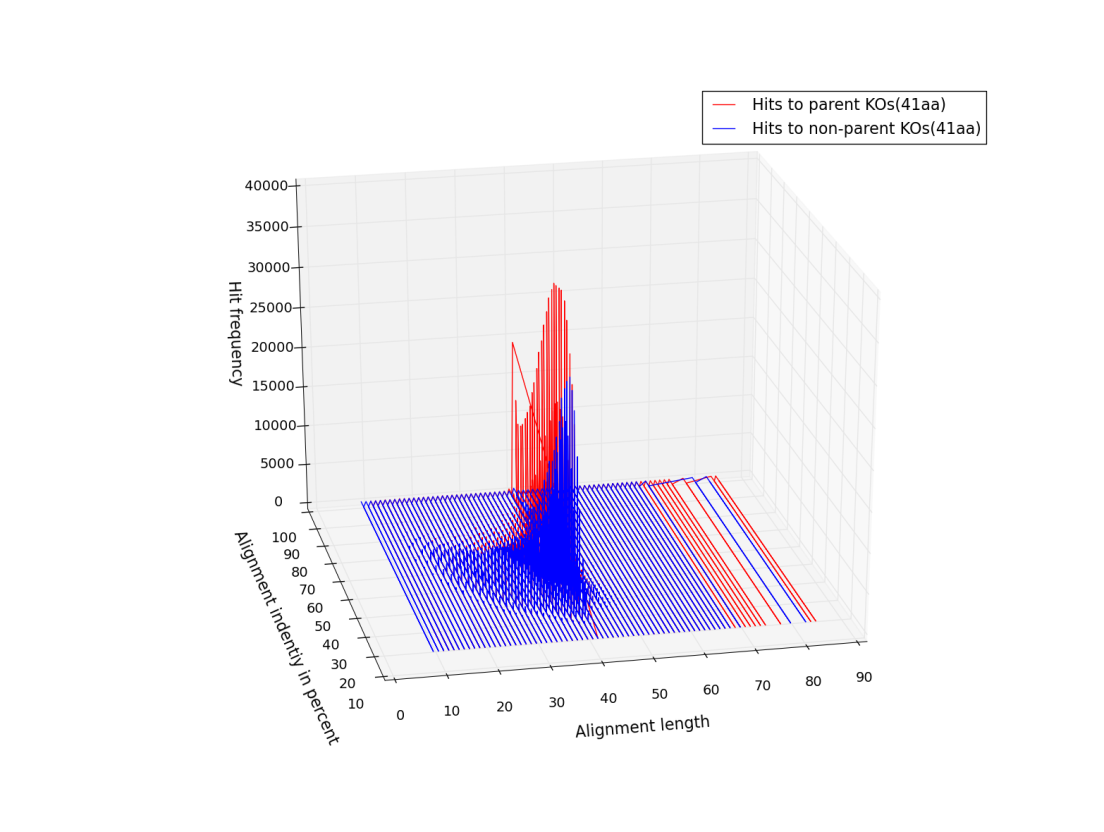  Figure S 19: 3D plot of alignment length, alignment-identity and frequency of hits to parent KO groups (Red) and non-parent KO groups (Blue). Length of peptides in the dataset is 41 amino acids. Significant overlap of blue and red graphs substantiates the absence of sequence similarity cutoffs to distinguish these two kinds of alignments |
| --- |

| 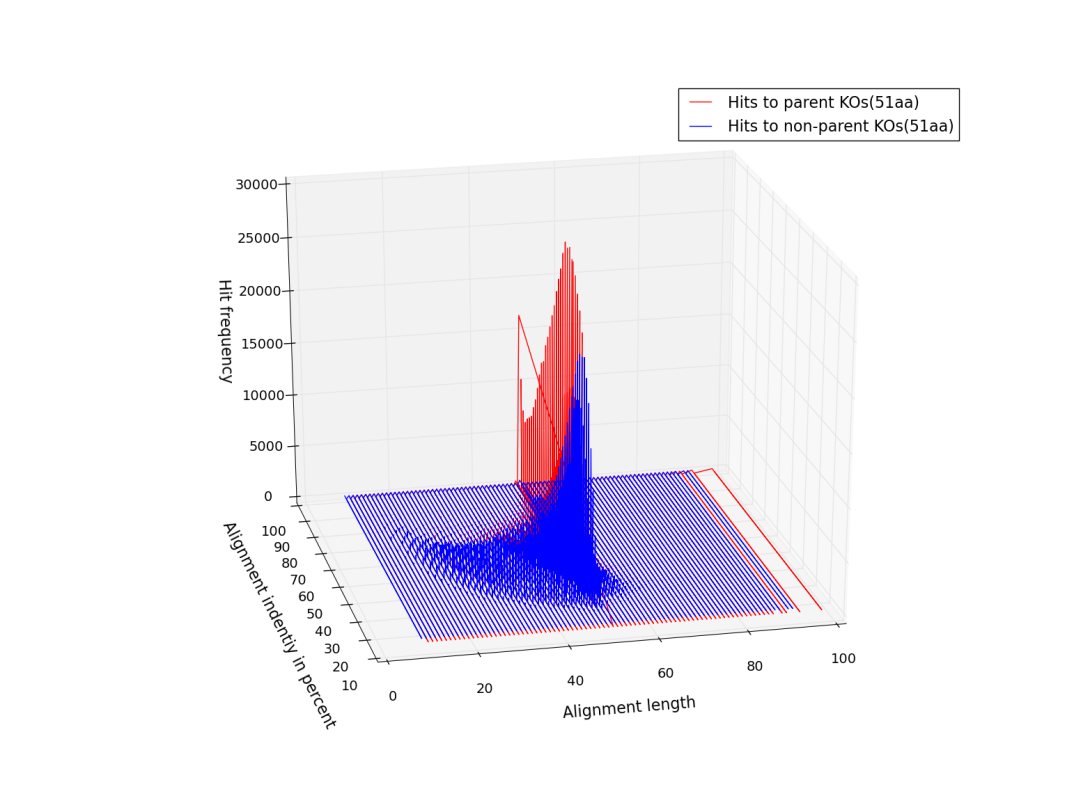  Figure S 20: 3D plot of alignment length, alignment-identity and frequency of hits to parent KO groups (Red) and non-parent KO groups (Blue). Length of peptides in the dataset is 51 amino acids. Significant overlap of blue and red graphs substantiates the absence of sequence similarity cutoffs to distinguish these two kinds of alignments |
| --- |

| 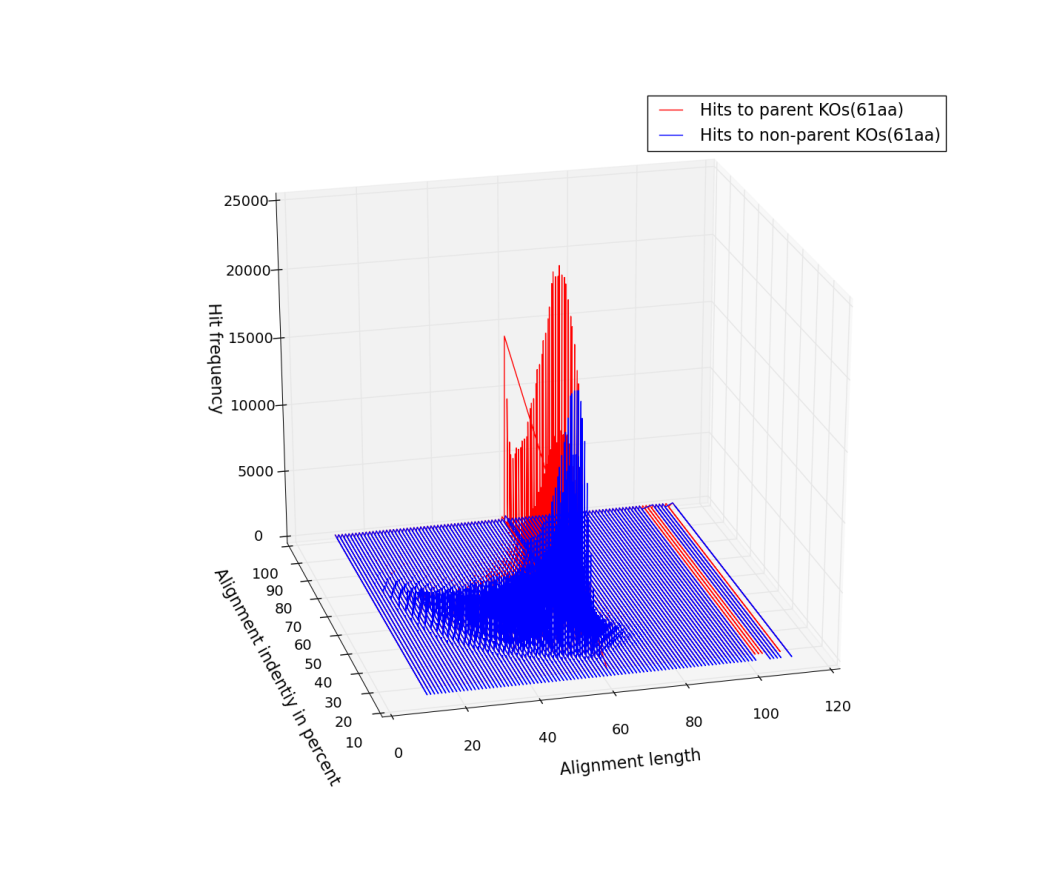  Figure S 21: 3D plot of alignment length, alignment-identity and frequency of hits to parent KO groups (Red) and non-parent KO groups (Blue). Length of peptides in the dataset is 61 amino acids .Significant overlap of blue and red graphs substantiates the absence of sequence similarity cutoffs to distinguish these two kinds of alignments |
| --- |

| 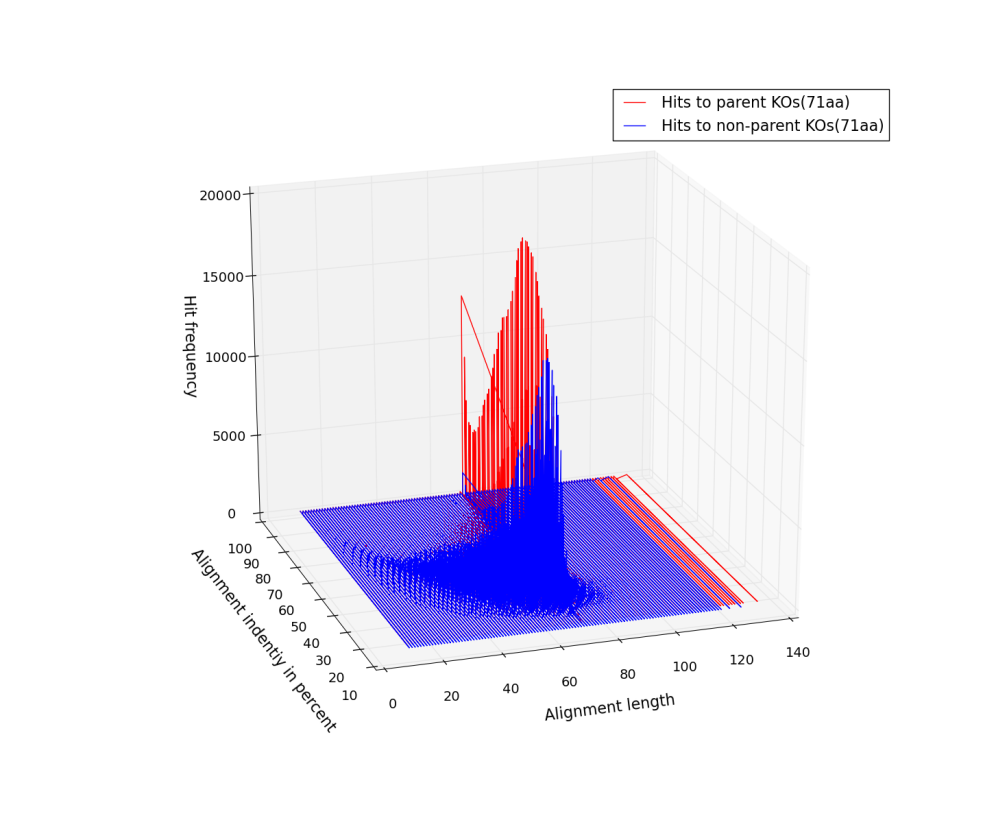  Figure S 22: 3D plot of alignment length, alignment-identity and frequency of hits to parent KO groups (Red) and non-parent KO groups (Blue). Length of peptides in the dataset is 71 amino acids. Significant overlap of blue and red graphs substantiates the absence of sequence similarity cutoffs to distinguish these two kinds of alignments |
| --- |

| 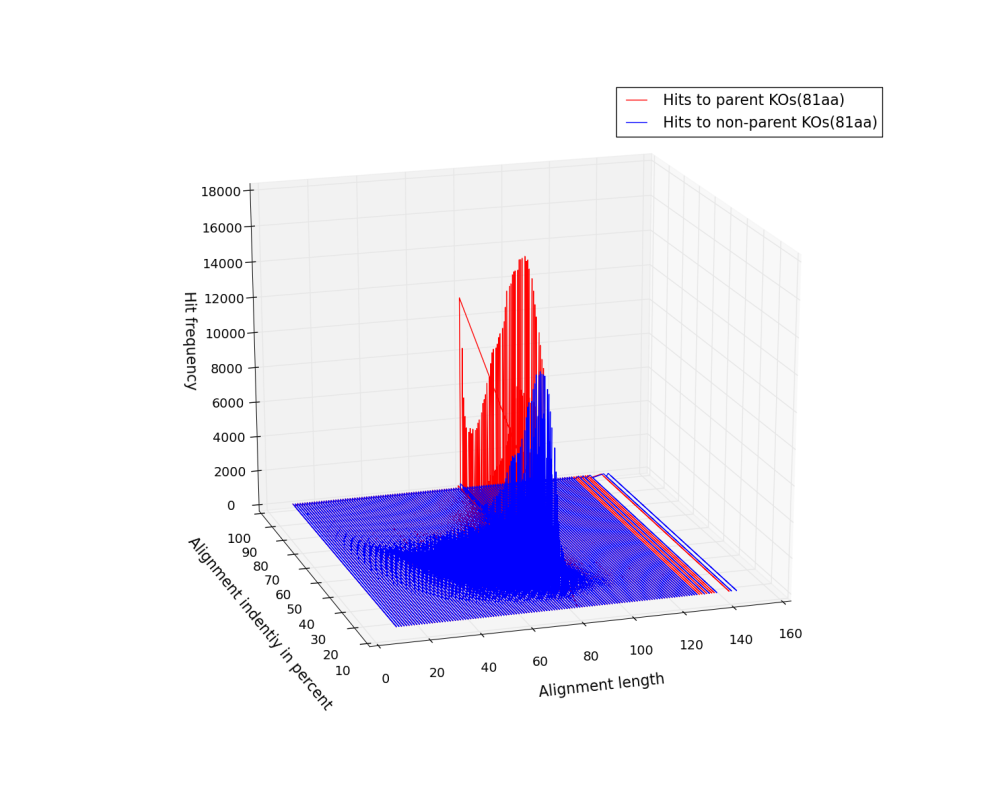  Figure S 23: 3D plot of alignment length, alignment-identity and frequency of hits to parent KO groups (Red) and non-parent KO groups (Blue). Length of peptides in the dataset is 81 amino acids. Significant overlap of blue and red graphs substantiates the absence of sequence similarity cutoffs to distinguish these two kinds of alignments |
| --- |

| 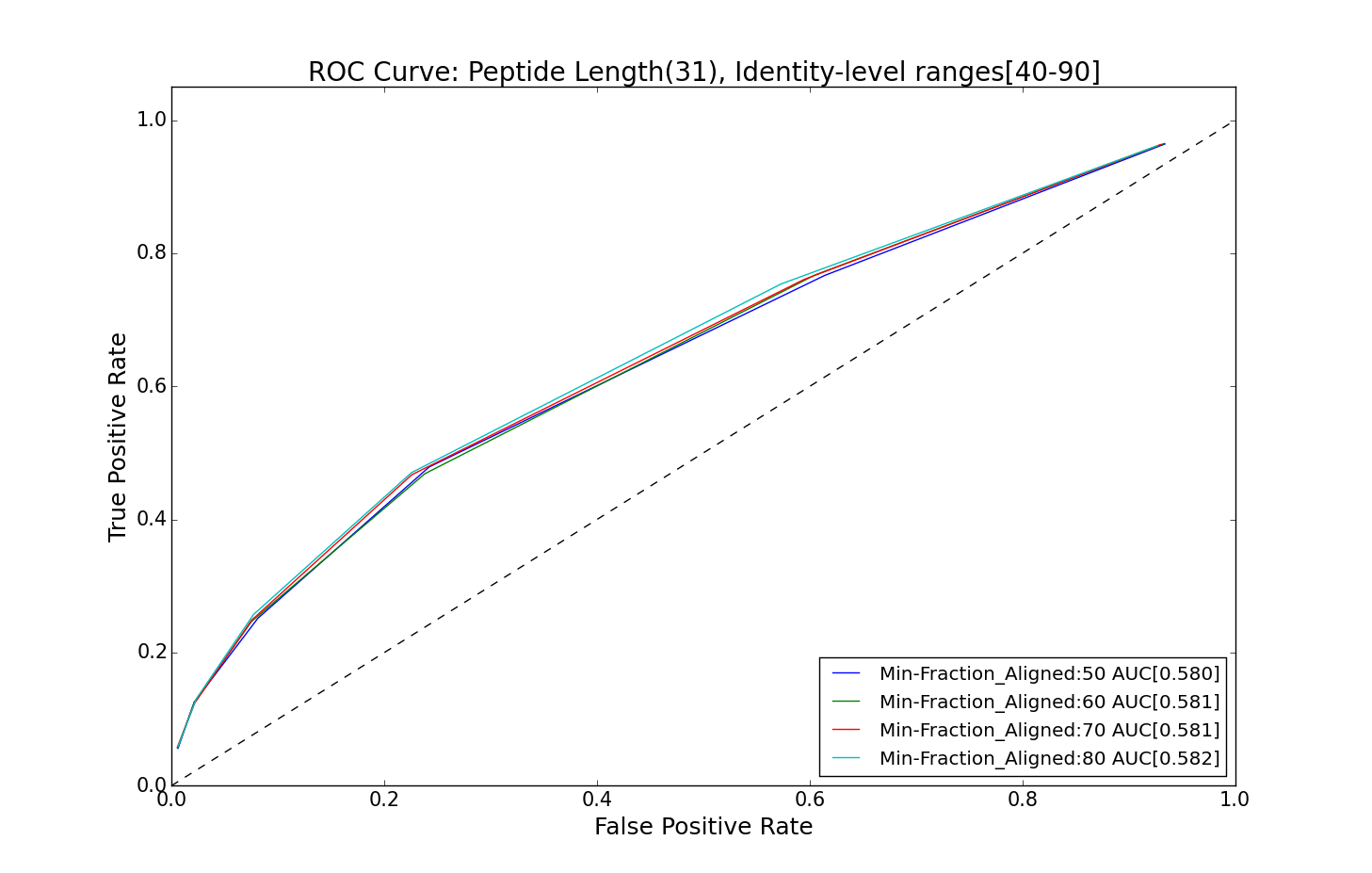A | 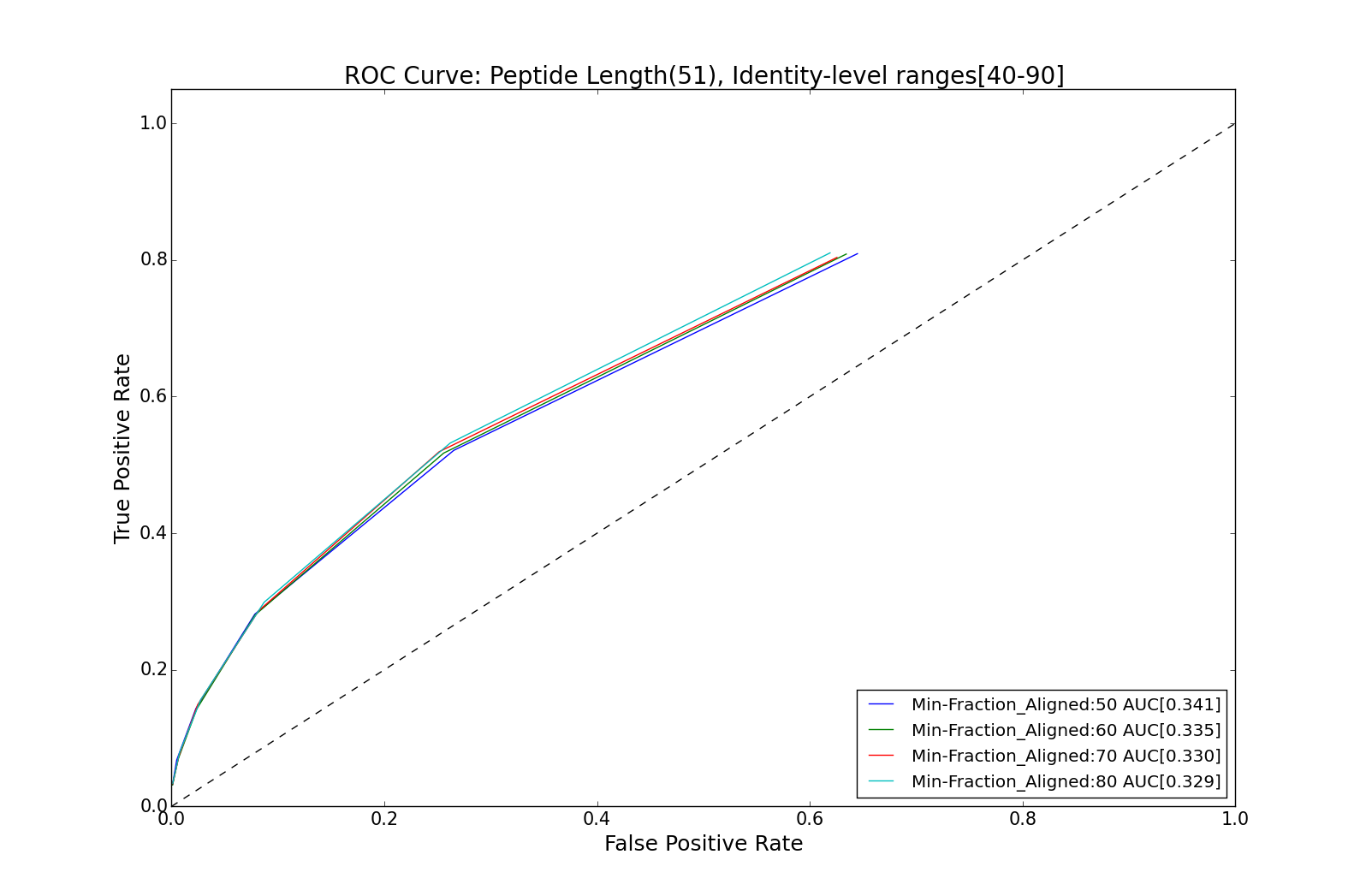B | 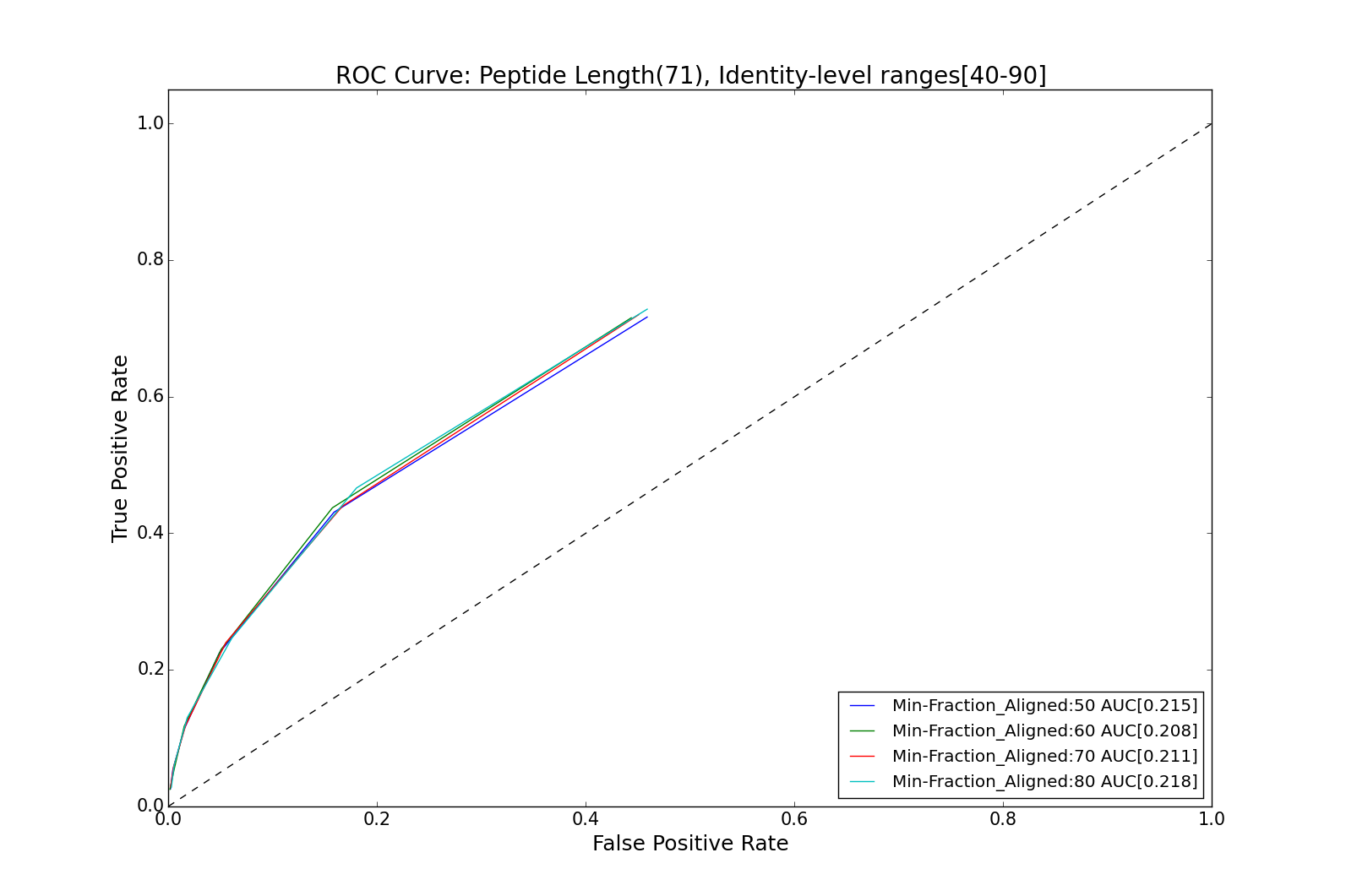C |
| --- | --- | --- |
| **Figure S 24:** Receiver Operating Characteristics curve reflecting success of alignment based assignment of functions to short peptides. Panel A-C reflects different lengths of peptides (31, 51 & 71aa). Multiple alignment parameters had been tested (identity ranged from 40% to 90% with increment of 10% and alignment-coverage ranged between 50-80%). | | |

| 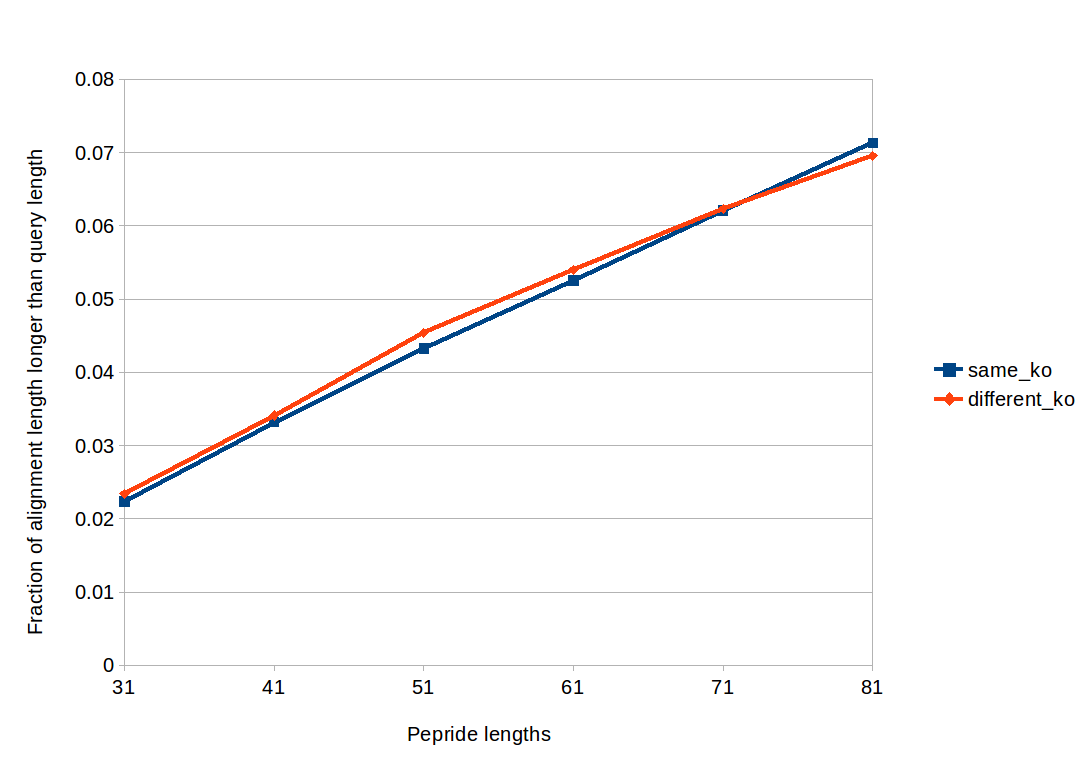  Figure S 25: Two lines representing the fraction of hits having alignment lengths longer than the query (peptide) length. Evidently blue (hits to same KO groups) and red lines (hits to different KO groups) show similar profiles. |
| --- |

| Figure S 26: Total members within a KO-group (log values) are plotted against median value of alignment identities. Only those alignments are considered when peptides (length 11) for dataset Type1.1 hits to members of their own KO-groups. Median alignment identities tend to decrease as number of members increases and high identity matches decreases as peptide length increases |
| --- |
| 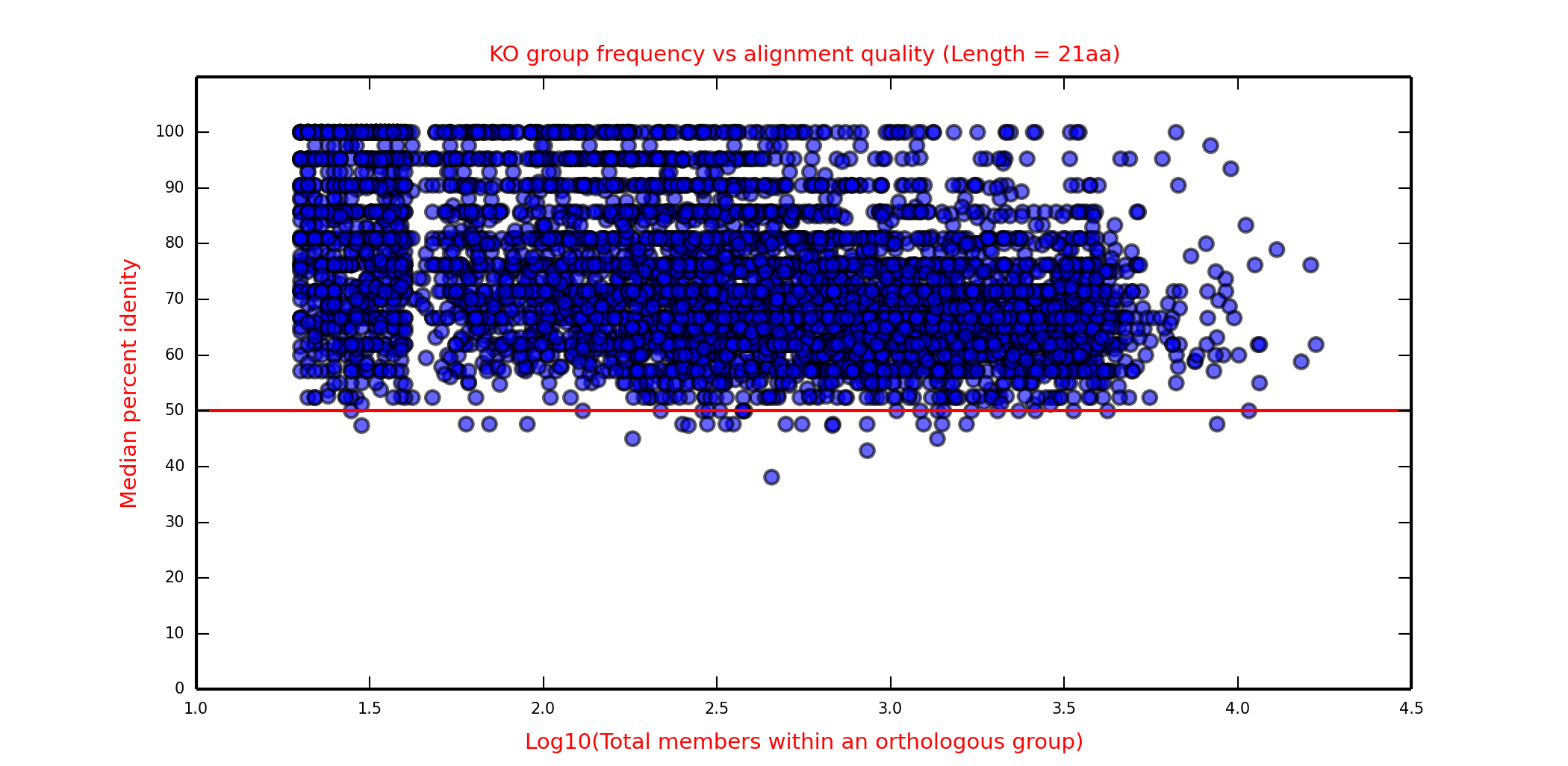  Figure S 27: Total members within a KO-group (log values) are plotted against median value of alignment identities. Only those alignments are considered when peptides (length 21) for dataset Type1.2 hits to members of their own KO-groups. Median alignment identities tend to decrease as number of members increases and high identity matches decreases as peptide length increases |
| 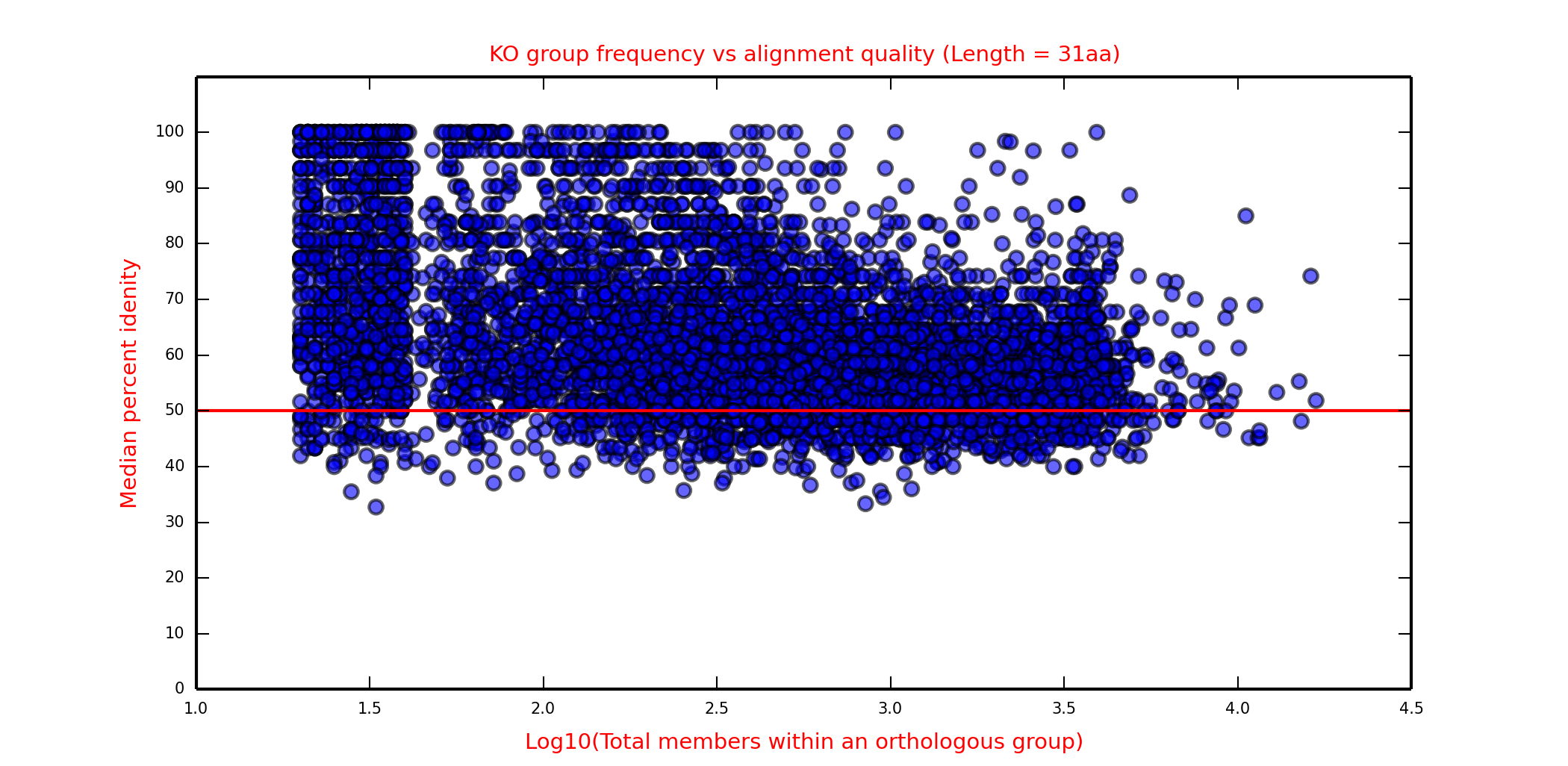  Figure S 28: Total members within a KO-group (log values) are plotted against median value of alignment identities. Only those alignments are considered when peptides (length 31) for dataset Type1.3 hits to members of their own KO-groups. Median alignment identities tend to decrease as number of members increases and high identity matches decreases as peptide length increases |
| 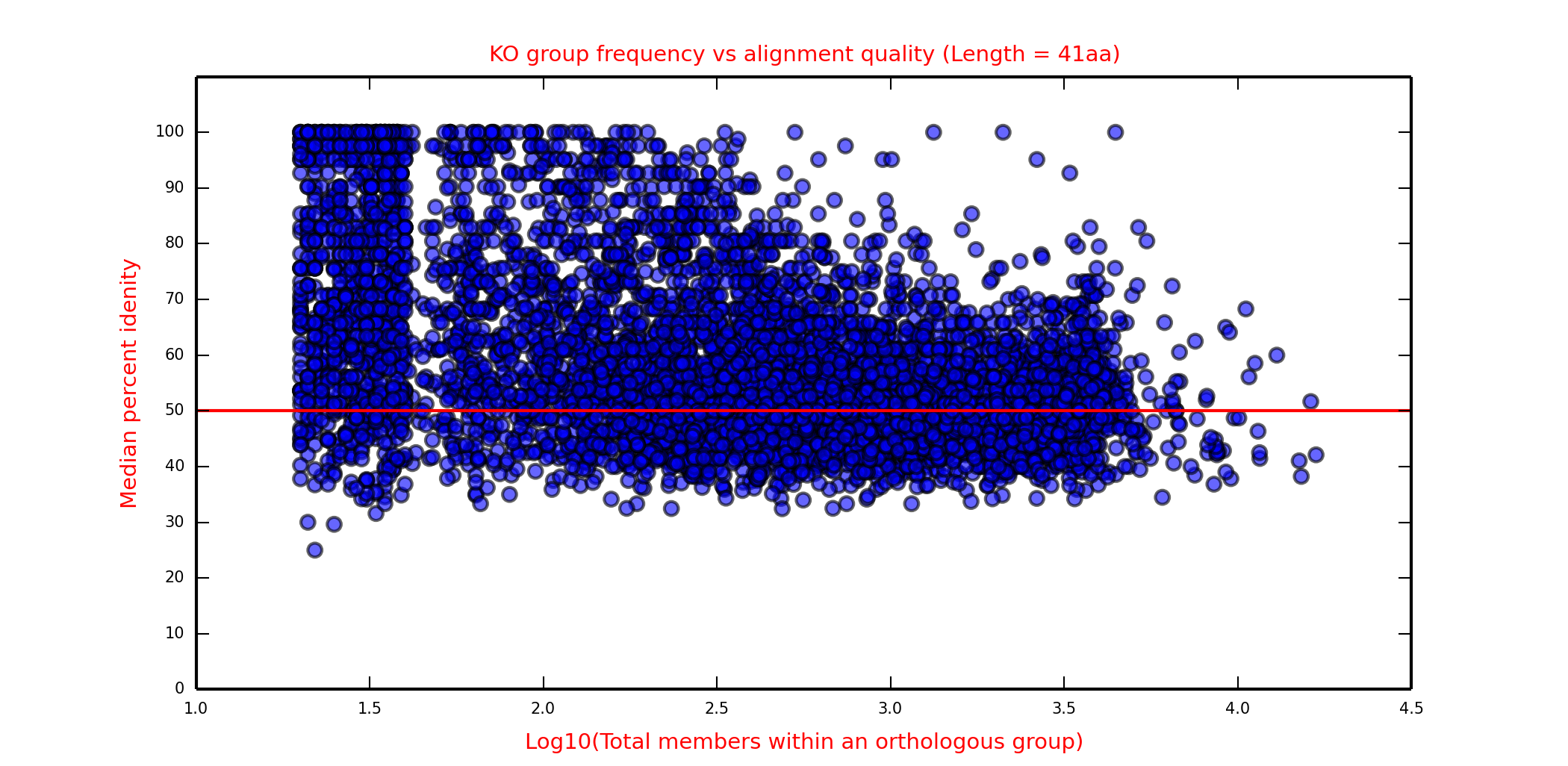  Figure S 29: Total members within a KO-group (log values) are plotted against median value of alignment identities. Only those alignments are considered when peptides (length 41) for dataset Type1.4 hits to members of their own KO-groups. Median alignment identities tend to decrease as number of members increases and high identity matches decreases as peptide length increases |
| 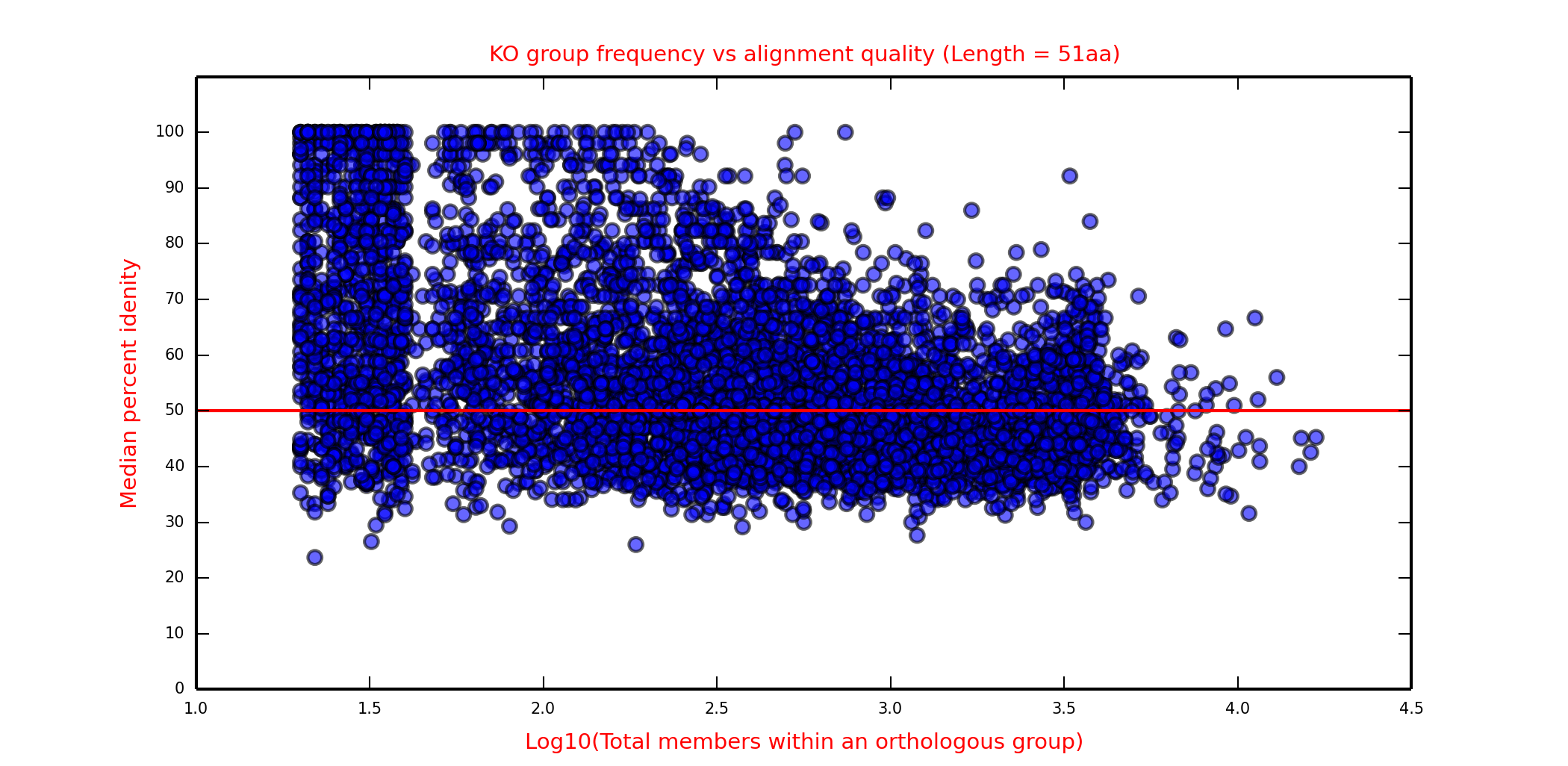  Figure S 30: Total members within a KO-group (log values) are plotted against median value of alignment identities. Only those alignments are considered when peptides (length 51) for dataset Type1.5 hits to members of their own KO-groups. Median alignment identities tend to decrease as number of members increases and high identity matches decreases as peptide length increases |
| 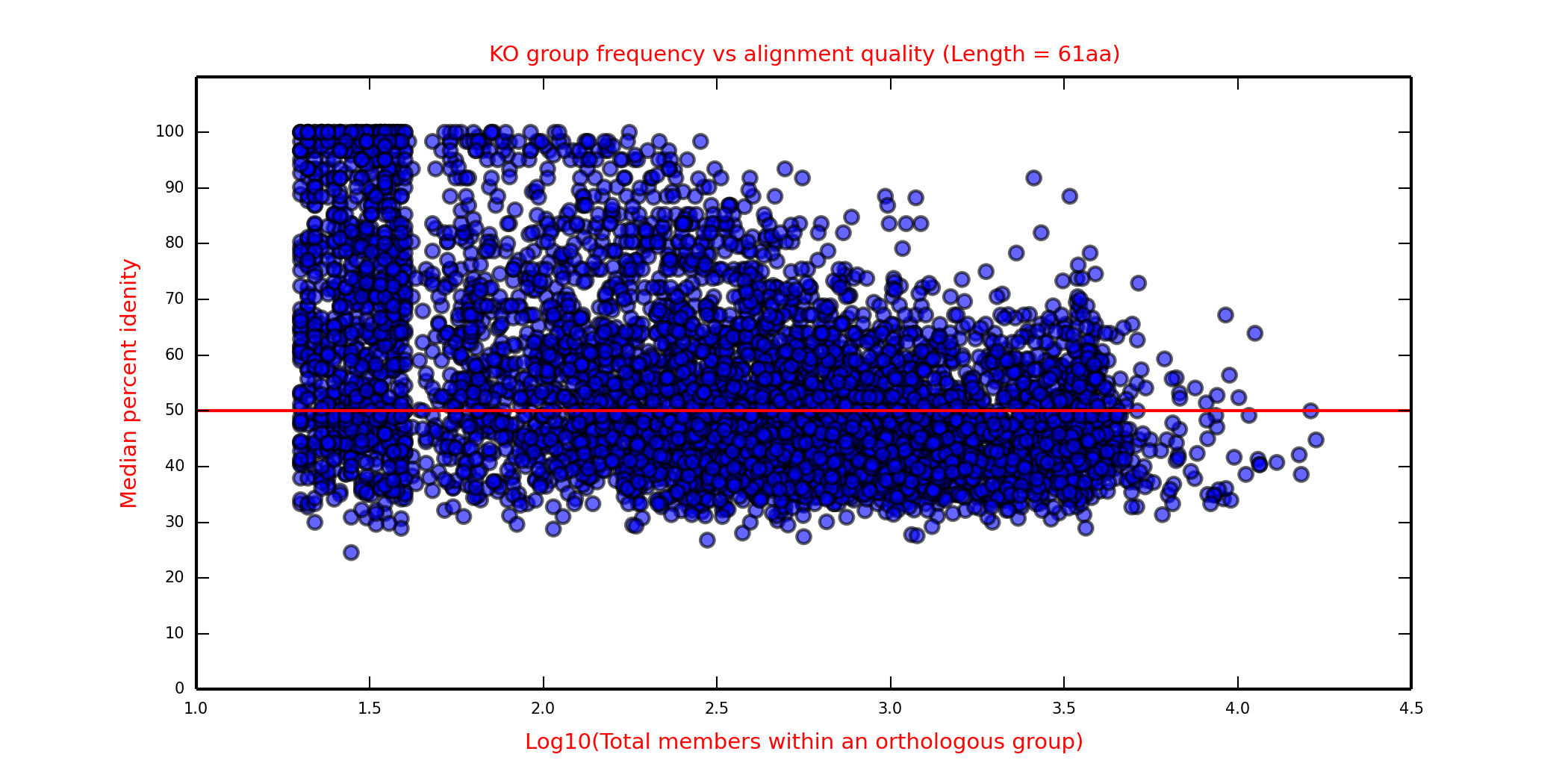  Figure S 31: Total members within a KO-group (log values) are plotted against median value of alignment identities. Only those alignments are considered when peptides (length 61) for dataset Type1.6 hits to members of their own KO-groups. Median alignment identities tend to decrease as number of members increases and high identity matches decreases as peptide length increases |
| 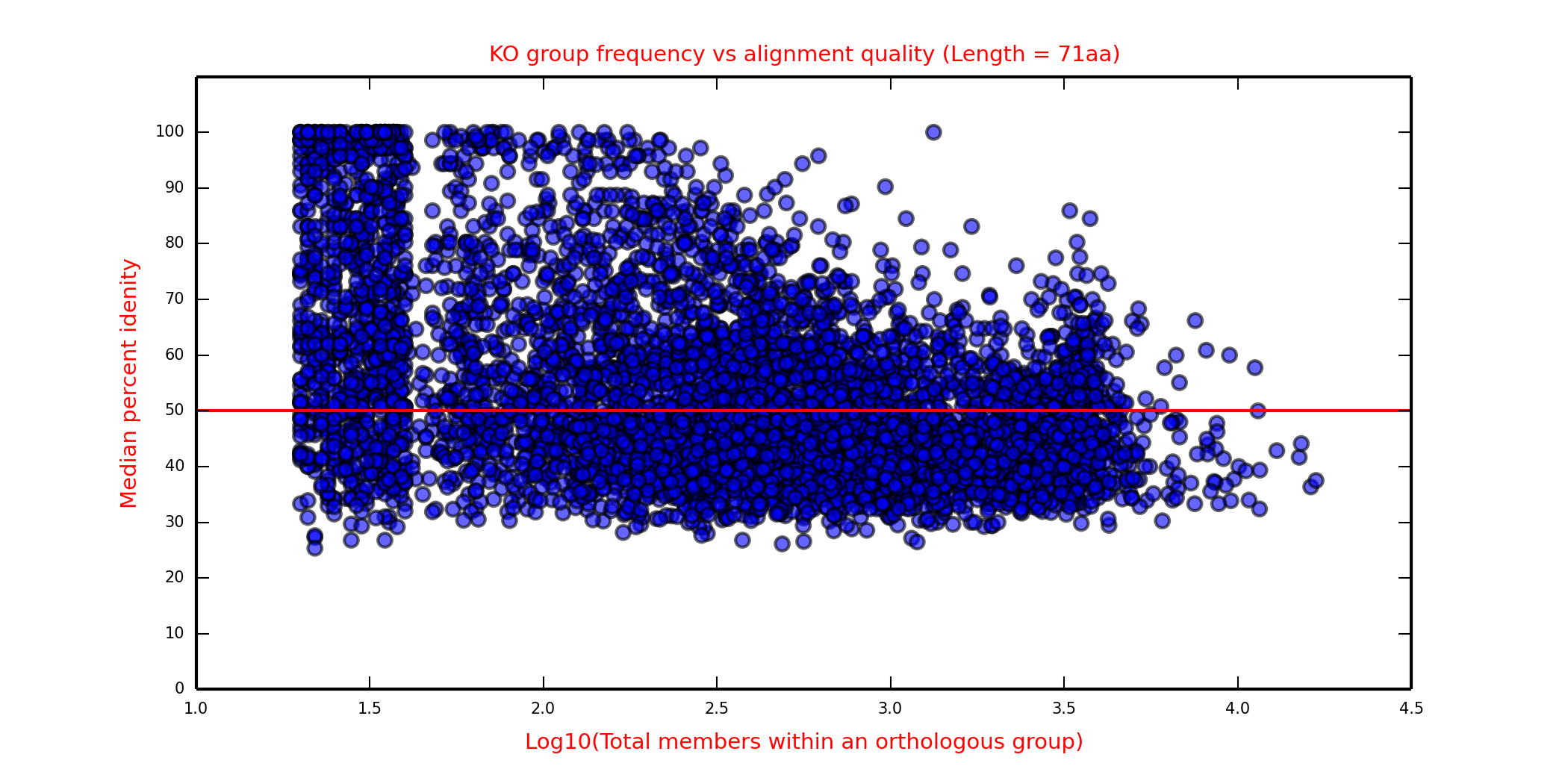  Figure S 32: Total members within a KO-group (log values) are plotted against median value of alignment identities. Only those alignments are considered when peptides (length 71) for dataset Type1.7 hits to members of their own KO-groups. Median alignment identities tend to decrease as number of members increases and high identity matches decreases as peptide length increases |
| 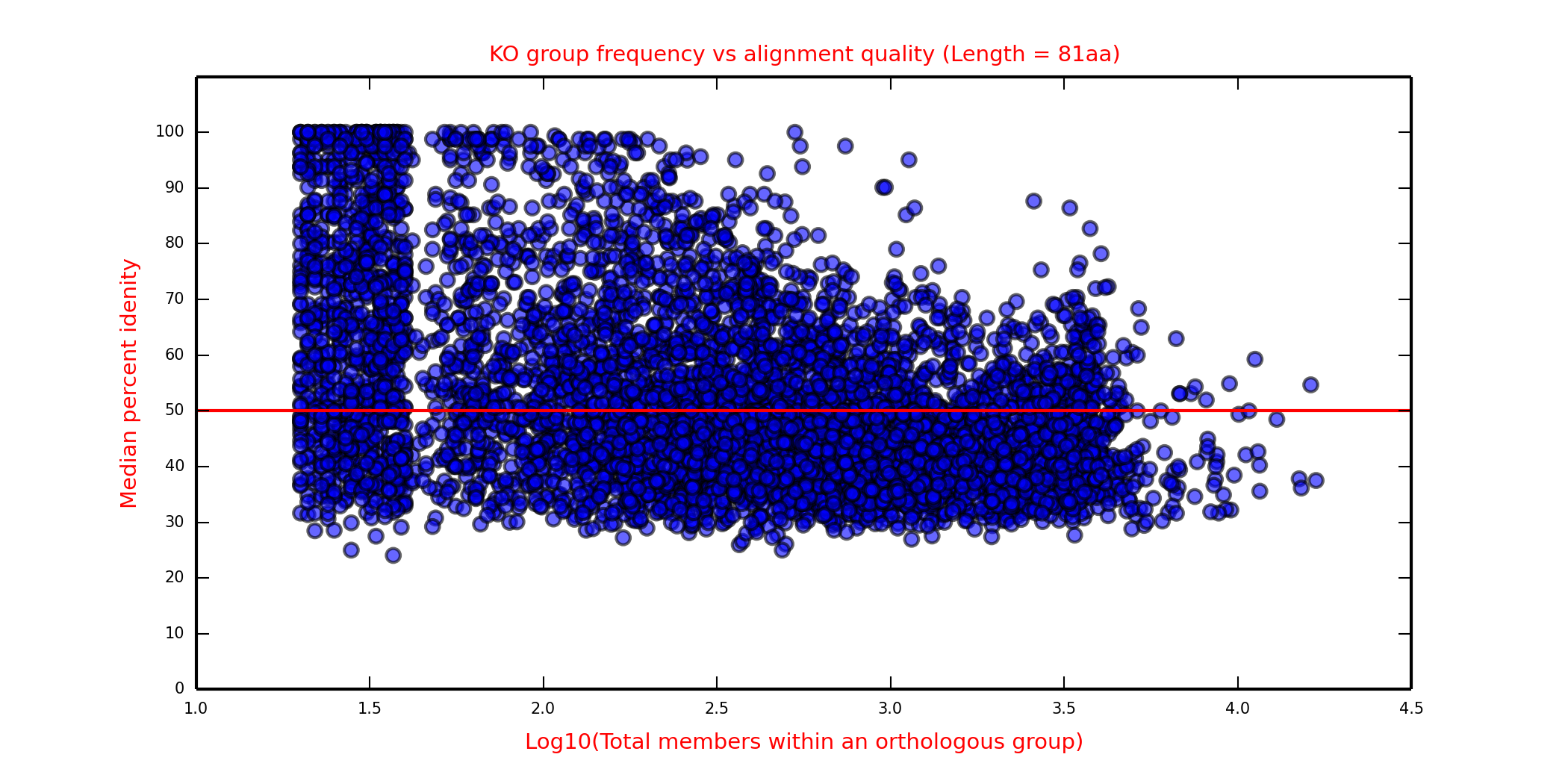  Figure S 33: Total members within a KO-group (log values) are plotted against median value of alignment identities. Only those alignments are considered when peptides (length 81) for dataset Type1.8 hits to members of their own KO-groups. Median alignment identities tend to decrease as number of members increases and high identity matches decreases as peptide length increases |

| 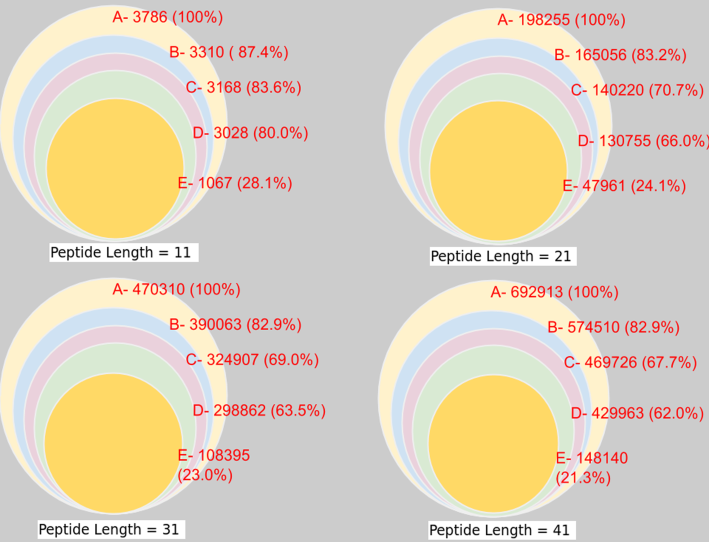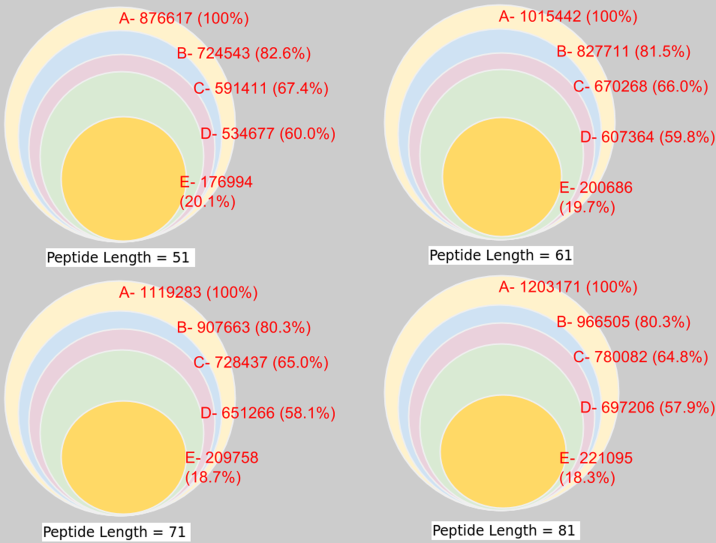  Figure S 34: Eight recursive circular diagrams (Test case1.1 to 1.8 BLAST output analyses) showing proportions of hits to non-parental KO-groups sharing same Enzyme Commission (EC) number, at different hierarchy levels. (A) Total number of hits to non-parental KO-group for which we could get EC-numbers of both test case peptides and ‘Reference set’ proteins. (B) Total number (fraction) of those hits sharing same 1^st^ level EC hierarchy. (C) Total number (fraction) of those hits sharing same 1^st^ and 2^nd^ level EC hierarchy. (D) Total number (fraction) of those hits sharing same 1^st^ , 2^nd^ and 3^rd^ level EC hierarchy. (E) Total number (fraction) of those hits sharing same EC-number (all 4 hierarchy levels). A majority of hits to non-parental KO-groups are restricted to the realm of the same EC hierarchy rather than being random in nature. |
| --- |

| 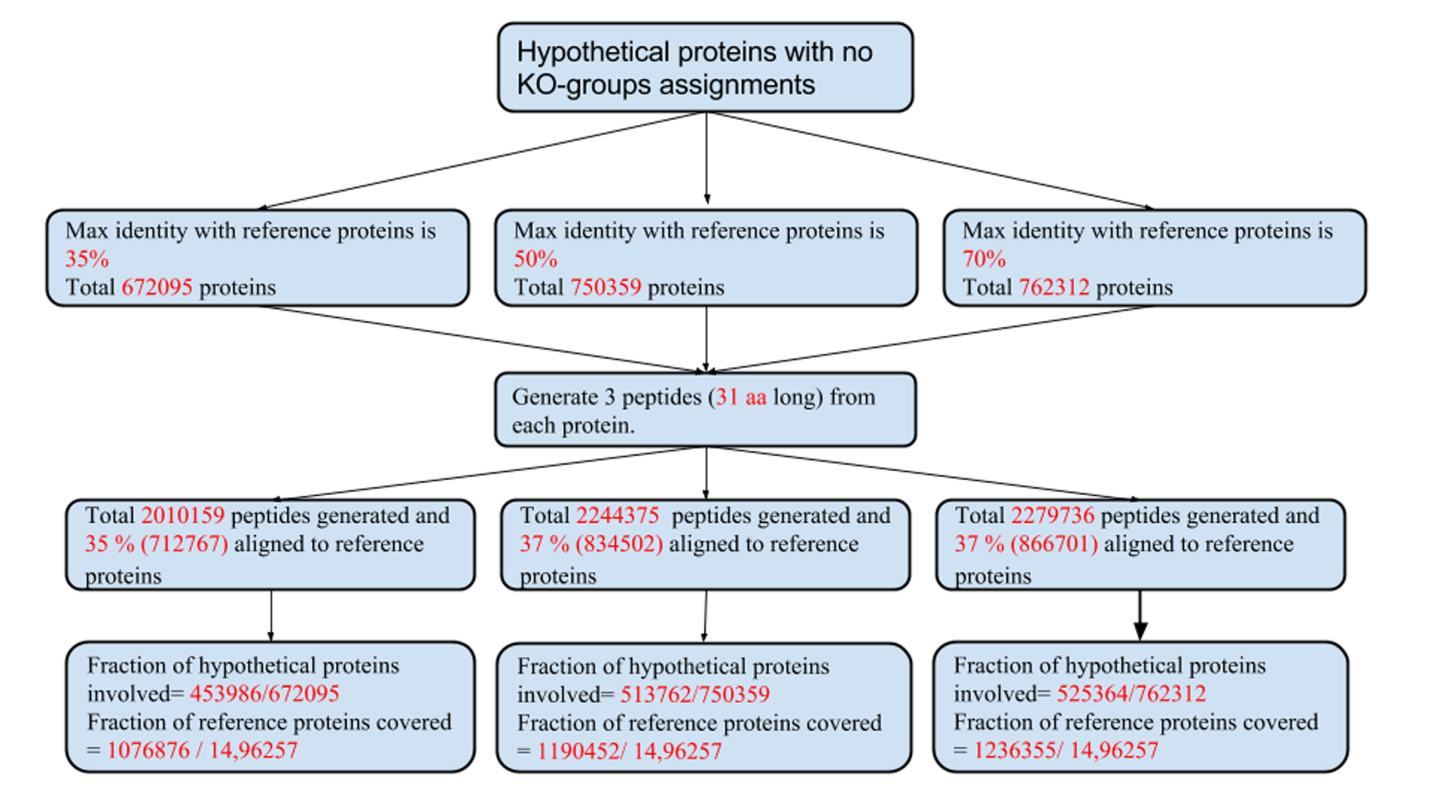  Figure S 35: A flown diagram to represent our protocol to generate the test set, which emulates the part of a metagenomic set which cannot be annotated with existing experimentally characterized proteins. Detailed behavior of such peptides is discussed in the result section of the manuscript. |
| --- |

| 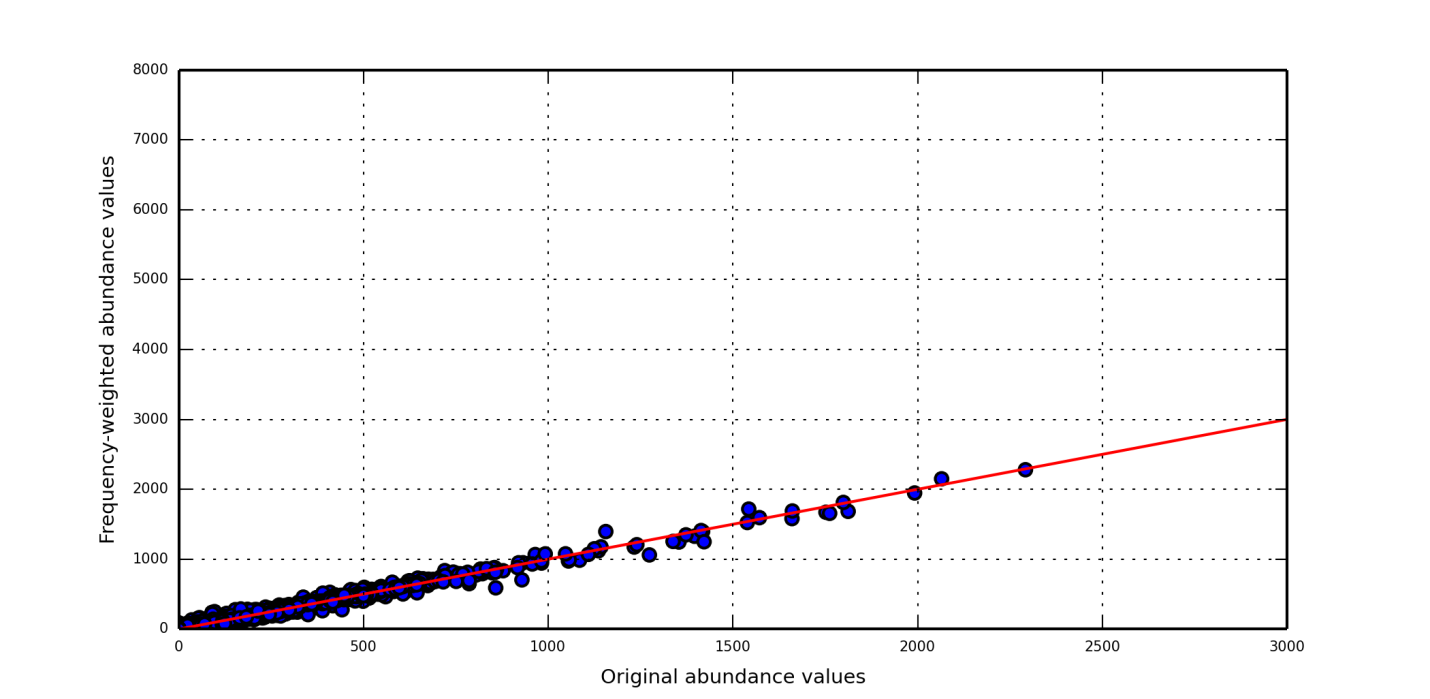  Figure S 36:’Frequency weighted count read’ method based abundance profile of ‘Select_4K_KO_Hits’ test set. A good correspondence (Pearson correlation 0.99) with real values is found. |
| --- |
| 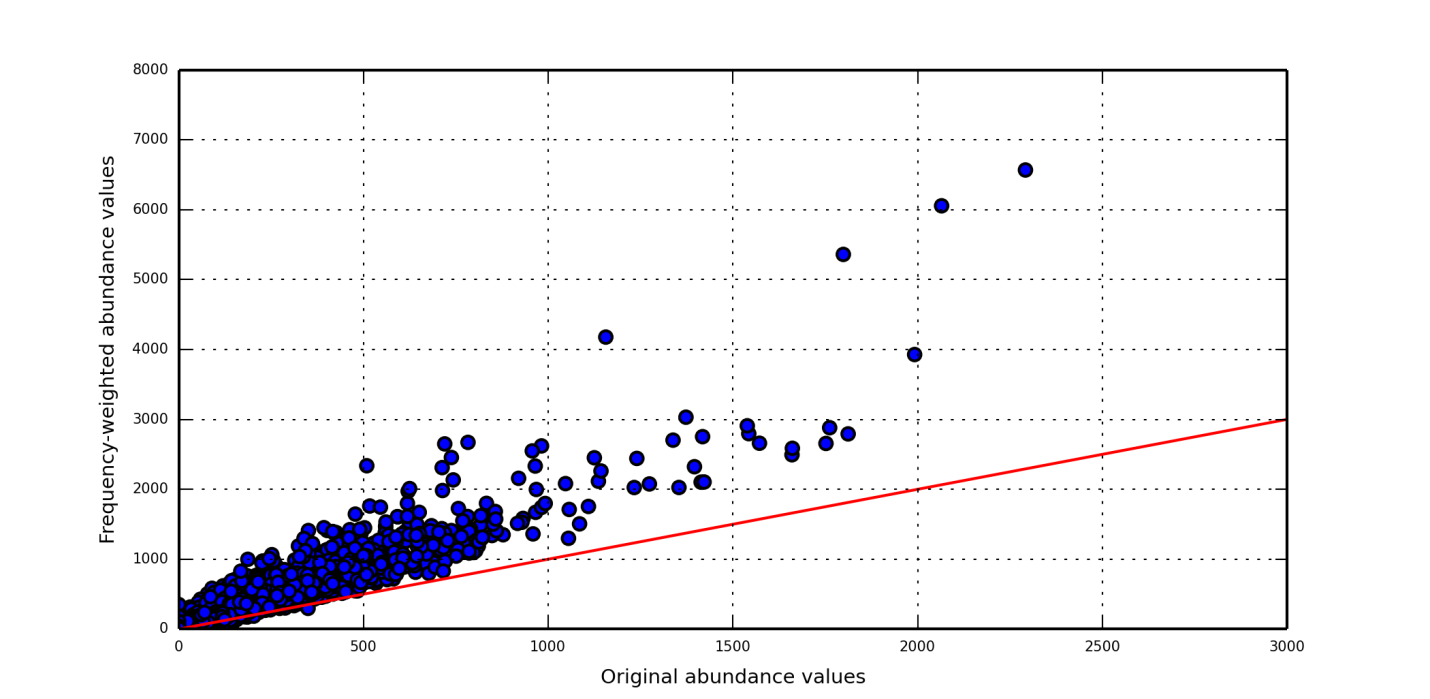  Figure S 37: ‘Frequency weighted count read’ method based abundance profile of data generated by adding ‘Select_4K_KO_Hits’ to the alignment results of ‘Test type2.3’ test set. It is evident that reads from uncharacterized proteins artificially boost the abundance of KO-groups. (Plot can be compared with Figure S35) |
| 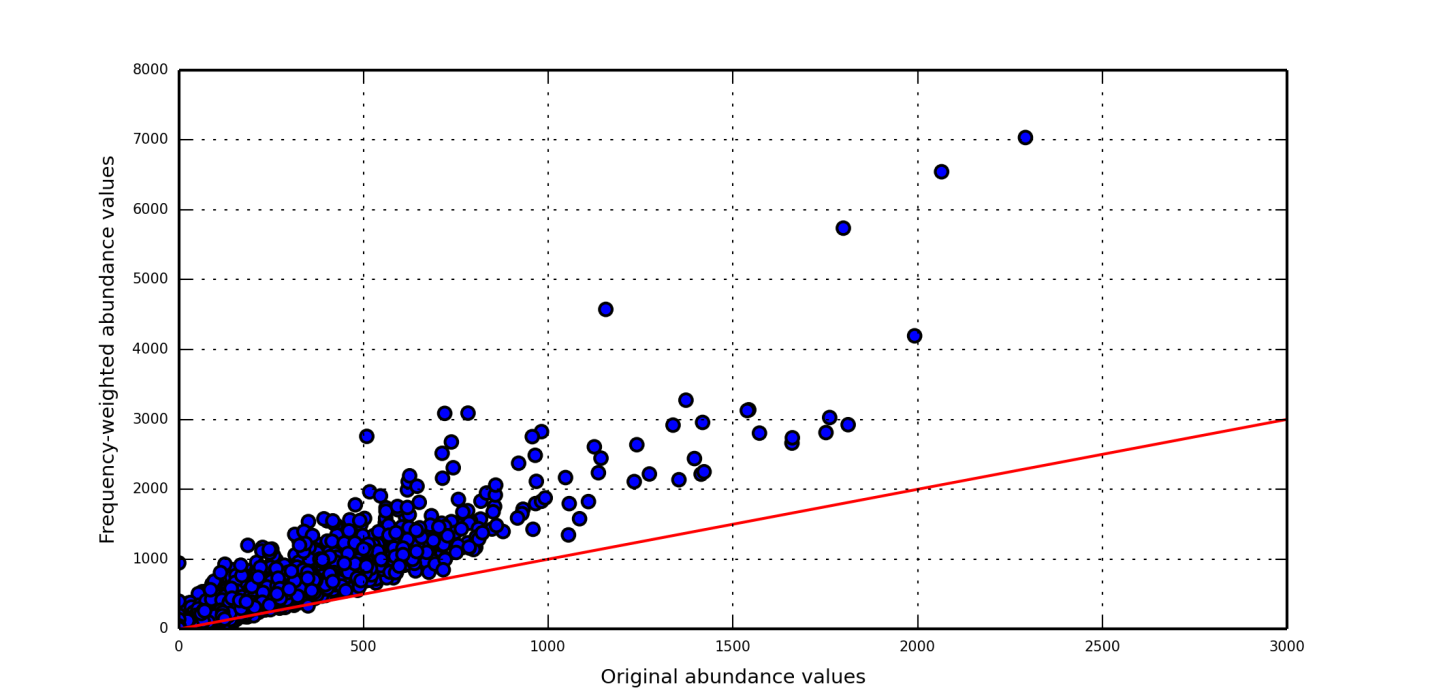  Figure S 38: ‘Frequency weighted count read’ method based abundance profile of data generated by adding ‘Select_4K_KO_Hits’ to the alignment results of ‘Test type2.2’ test set. It is evident that reads from uncharacterized proteins artificially boost the abundance of KO-groups. (Plot can be compared with Figure S35) |
| 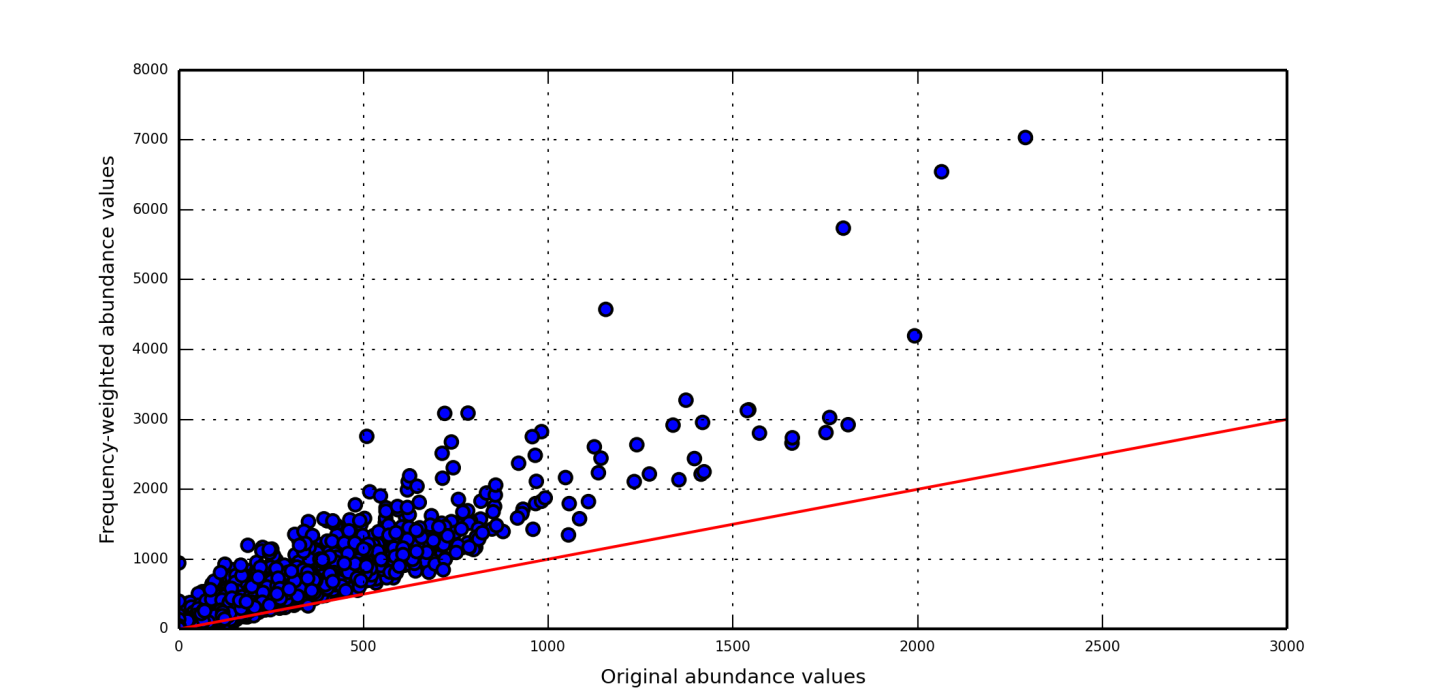  Figure S 39: ‘Frequency weighted count read’ method based abundance profile of data generated by adding ‘Select_4K_KO_Hits’ to the alignment results of ‘Test type2.1’ test set. It is evident that reads from uncharacterized proteins artificially boost the abundance of KO-groups. (Plot can be compared with Figure S35) |

| 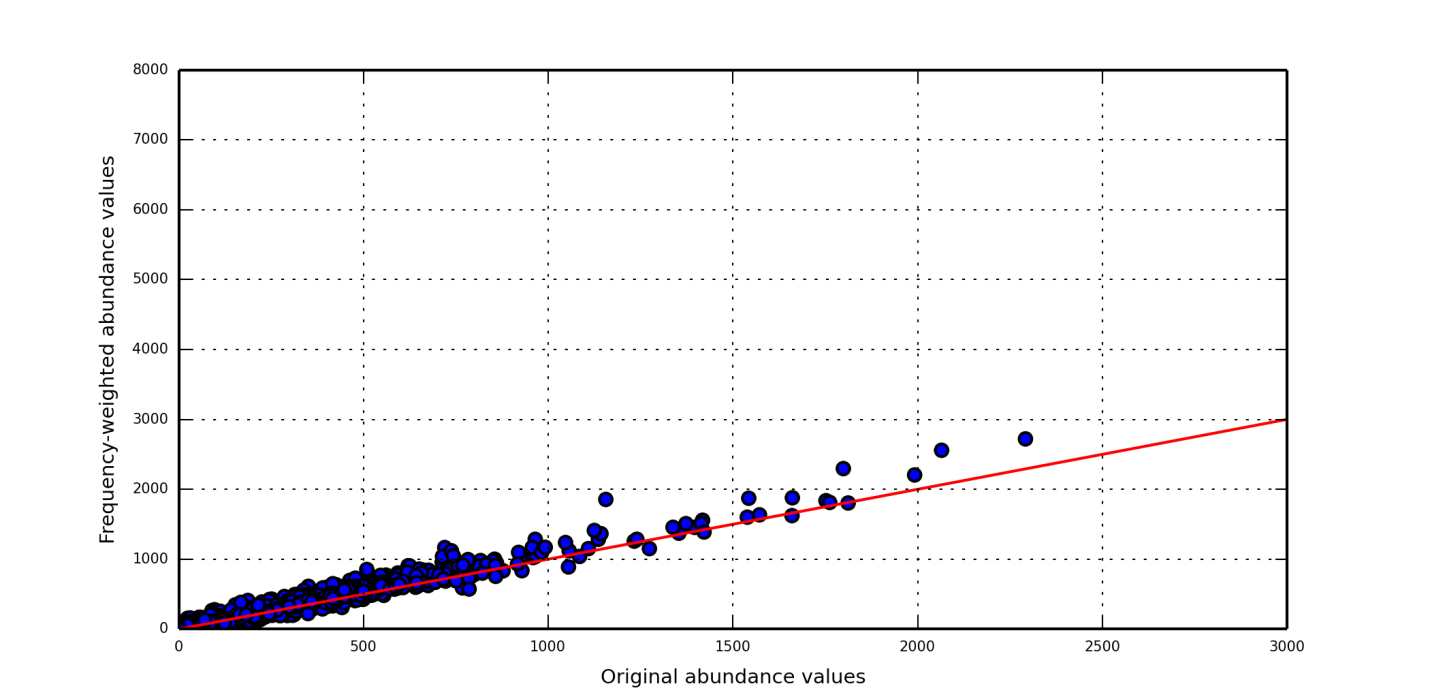  Figure S 40: Correction of artificial abundance boost by adding ‘random-hit filtering module’ in our Frequency weight read count protocol. New abundance profile of the test data used in Figure S36 (plot can be directly compared to the plot in Figure S36) |
| --- |
| 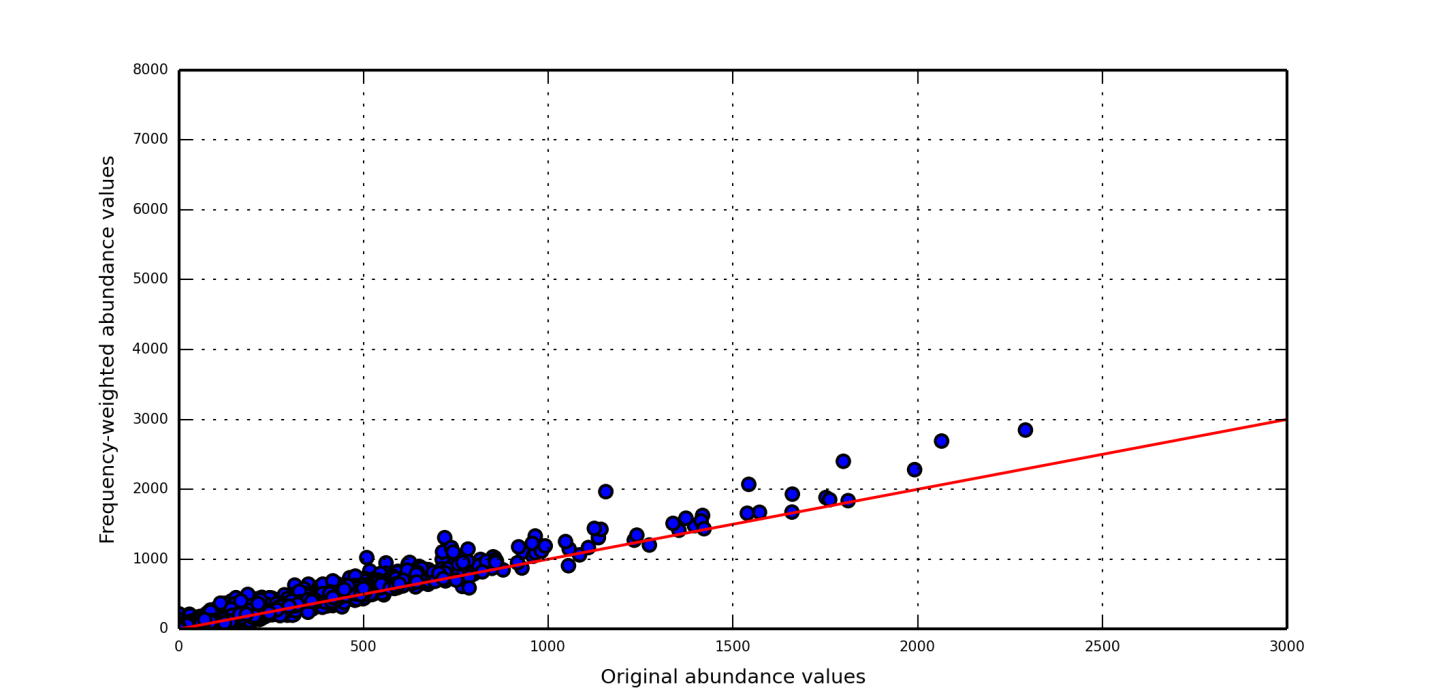  Figure S 41: Correction of artificial abundance boost by adding ‘random-hit filtering module’ in our Frequency weight read count protocol. New abundance profile of the test data used in Figure S37 (plot can be directly compared to the plot in Figure S37) |
| 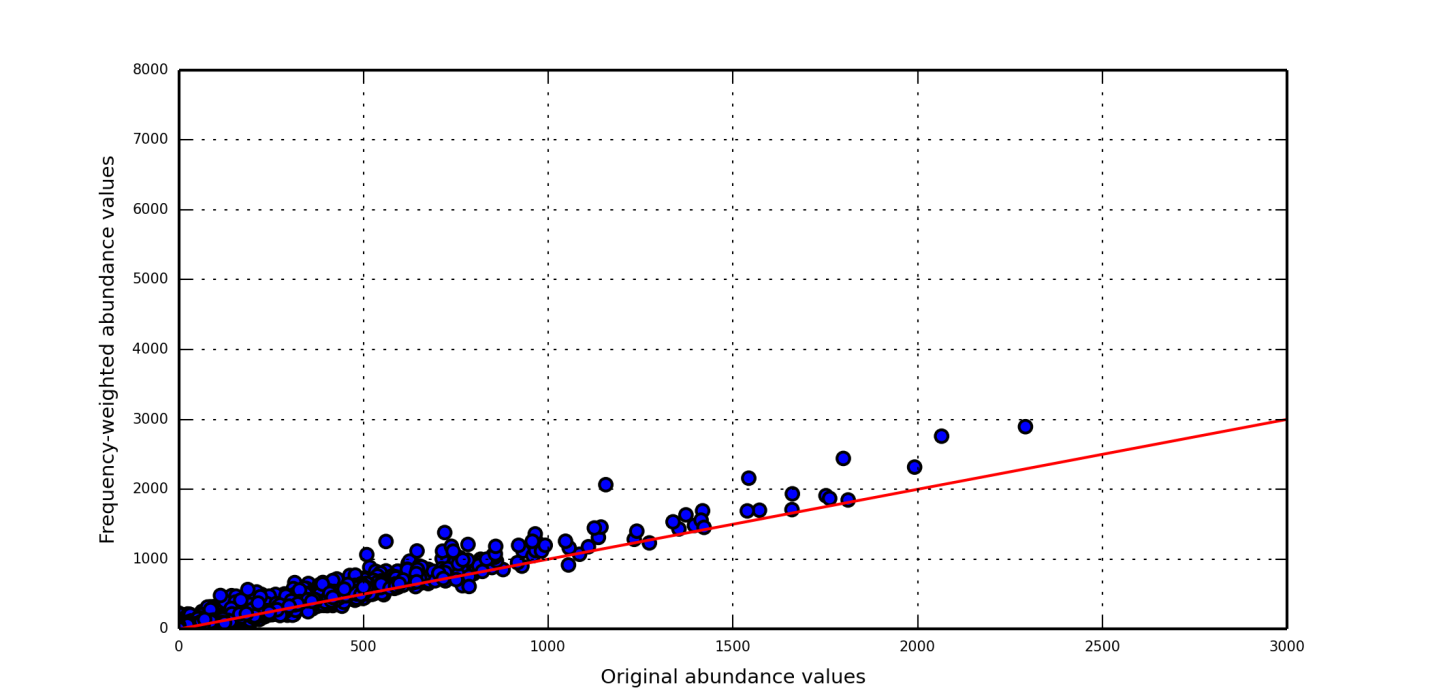  Figure S 42: Correction of artificial abundance boost by adding ‘random-hit filtering module’ in our Frequency weight read count protocol. New abundance profile of the test data used in Figure S38 (plot can be directly compared to the plot in Figure S38) |

| 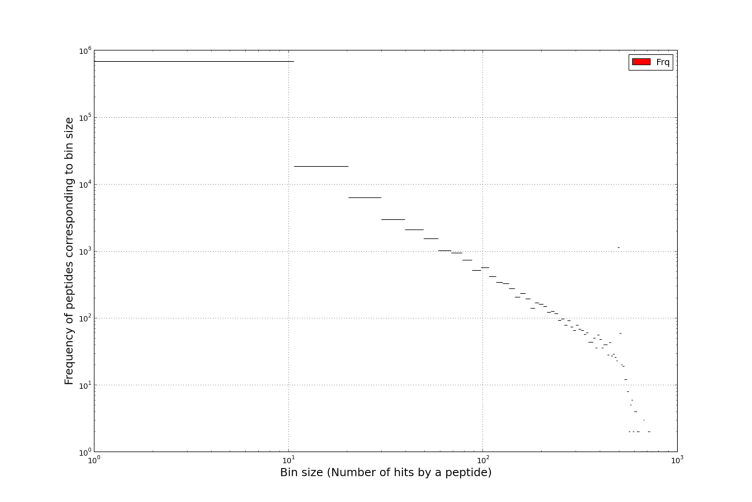  Figure S 43: Per peptide hit pattern of peptides from ‘Test case type 2.1’. The majority of the peptides have 0 to 10 hits. |
| --- |
| 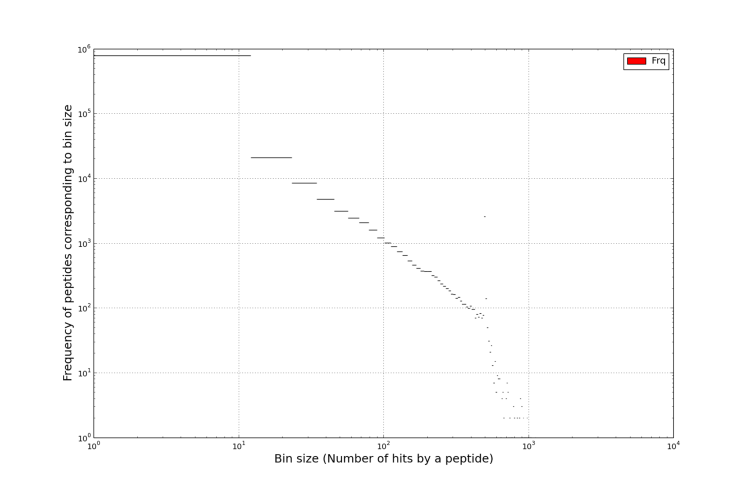  Figure S 44: Per peptide hits pattern of peptides from ‘Test case type 2.2’. The majority of the peptides have 0 to 10 hits |
| 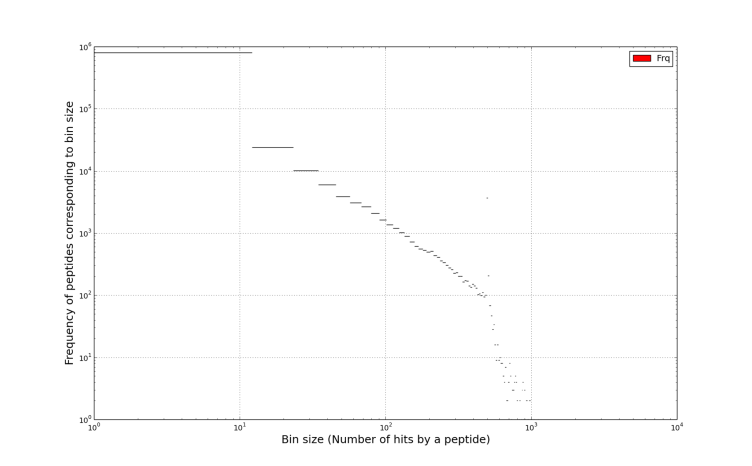  Figure S 45: Per peptide hits pattern of peptides from ‘Test case type 2.3’. The majority of the peptides have 0 to 10 hits |

# Data files and codes description

Input data and codes used in this paper are hosted at (<http://cage.unl.edu/DataPeptide>). A brief description of the shared files is below.

1. Protein sequences used in the present work were obtained from M5nr database.
2. **BacteriaKEGG_KO_Available.fa:** Bacterial proteins having KEGG-orthologous group assignments. Total 1496257 such proteins were derived from M5nr database.
3. **DataPeptide/TestCase_Type1/Step4.txt:** This is a table where the rows are M5nr protein-id and the columns carry multiple features of corresponding proteins (e.g KEGG_ID, KO-value, Pathway)
4. **DataPeptide/TestCase_Type1/SimulateRead.py:** This is the python script used to generate the Type1 data set. It requires “Step4.txt” and “BacteriaKEGG_KO_Available.fa” as input.
5. **TestCase_Type1:** Folder “TestCase_Type1” contains simulated peptides of ‘type-1’ (check METHODS section for details). It also contains the “BLASTP” alignment of ‘type-1’ peptides to proteins from “BacteriaKEGG_KO_Available.fa”. Simulated peptides and their “BLASTP” alignments are in subfolders K11-K81, (numerical value corresponds to the length of short-peptide)
6. **DataPeptide/****TestCase_Type2/Peptides_From_HypotheticalProteins:** Peptides derived from hypothetical proteins.
7. **DataPeptide/TestCase_Type2/BlastOutput_SelectedHits_4000_KO:** “BLASTP” output of “Selected_4K” peptide group (check METHODS section for details).
8. **DataPeptide/TestCase_Type2/BlastOutput_Combine_ID_N:** “BLASTP” output of peptides derived from hypothetical proteins (maximum identity with reference proteins is “N”, check METHODS section for details).
9. **DataPeptide/TestCase_Type2/Run_ID_N:** Run this shell script to calculate “filtered frequency weighted” abundance profiles of protein families.
10. **DataPeptide/TestCase_Type2/AbundanceSimulatedData_filter_Step*.py:** Codes used to generate the abundance prediction of “Test case_Type2” peptides
11. **DataPeptide/TestCase_Type2/DataFrq_*:** Frequency weighted protein-abundance profiles of Test case 2.1,2.2 and 2.3 are shared. For the same test cases, outputs of filter-enabled frequency weighting protocol are also shared. DataFrq_* format: [ 1^st^ column: KO names; 2^nd^ column: real read count of KOs and 3^rd^ column: Frequency weighted read count of KOs]
12. **DataPeptide/TestCase_Type3/ProteinID_PartOfStudy:** This file has IDs of proteins used to generate our Test Case_Type3. (Check METHODS section for details).
13. **DataPeptide/****TestCase_Type3/Peptides:** This folder has set of peptides (total 5412049) derived from 180510 proteins listed in “ProteinID_PartOfStudy”.
14. **DataPeptide/TestCase_Type3/Simulated_KO_Frq:** Frequency or total read count of each KEGG-orthologous group in Test Case_Type3.
15. **DataPeptide/TestCase_Type3/Peptides_BlastP:** BLASTP alignment results of “TestCase_Type3/Peptides”.
16. **DataPeptide/TestCase_Type3/Peptides_BlastP/AbundanceSimulatedData_Step*.py :** Codes used to generate the abundance prediction of “Test case_Type3” peptides.
17. **DataPeptide/TestCase_Type3/Peptides_BlastP/DataFrq_Predicted_ReadCount.tab:** Frequency weighted method is used to predict read counts on “Test case_Type3” peptides.
